# Avoidance of cardiac toxicity during the application of immune checkpoint inhibitors: a safety systematic review combining network meta-analysis and pharmacovigilance study

**DOI:** 10.3389/fimmu.2026.1919841

**Published:** 2026-08-31

**Authors:** Han Chen, Yongqi Shan, Hanfang Xu, Keer Xuan, Tianshu Ren, Qingchun Zhao

**Affiliations:** 1 Department of Clinical Pharmacy, General Hospital of Northern Theater Command, Shenyang, Liaoning, China; 2 Department of General Surgery, General Hospital of Northern Theater Command, Shenyang, Liaoning, China; 3 Department of Clinical Pharmacy, Shenyang Pharmaceutical University, Shenyang, Liaoning, China

**Keywords:** cardiac toxicity, disproportionality, immune checkpoint inhibitors, network meta-analysis, safety

## Abstract

**Background:**

Although immune checkpoint inhibitor (ICI) therapy has revolutionized cancer treatment, the incidence of cardiac toxicity associated with ICIs remains unclear. This study aimed to evaluate the cardiac toxicity risks associated with ICI and identify the most common types of cardiac toxicity caused by ICI. A further objective was to determine the types of ICI that require the most monitoring of cardiac toxicity through systematic review and network meta-analysis, assisted by pharmacovigilance studies.

**Design:**

Systematic review and network meta-analysis, complemented by a pharmacovigilance study of the FAERS database.

**Data source and methods:**

A systematic search of electronic databases up to November 2025 was conducted. Randomized controlled trials (RCTs) were eligible if they compared ICIs with appropriate controls without restrictions. Data were analyzed using random-effects pairwise and network meta-analyses to evaluate cardiac toxicity. Disproportionality analysis was performed using FAERS data from 2011 to 2026 (Q1), employing PRR, ROR, IC, and EBGM to quantify the risk and incidence of cardiac toxicity associated with ICIs.

**Results:**

In total, 135 RCTs were included in the network meta-analysis. Pairwise meta-analysis demonstrated that ICIs can induce cardiac toxicity in four key manifestations, which were selected through pairwise meta-analysis: acute myocardial infarction, cardiac arrest, myocarditis, and ventricular tachycardia. Network meta-analysis indicated that both PD-1/PD-L1 monotherapy and combination therapies present a higher risk of cardiac toxicity. The risks associated with PD-1 and PD-L1 agents were relatively elevated when used as monotherapies. Furthermore, combinations of PD-1 (nivolumab, pembrolizumab) and PD-L1 (avelumab) with CTLA-4 or other small molecule targeted inhibitors were associated with increased risk of the four aforementioned cardiac toxicities. Disproportionality analysis revealed that both PD-1 and PD-L1 inhibitors carry risks of myocarditis, and the combination of bevacizumab, relatlimab, and ipilimumab may cause increased cardiac toxicity.

**Conclusions:**

ICIs, particularly PD-1/PD-L1 inhibitors, increase the risk of cardiac toxicity. PD-1 (nivolumab, pembrolizumab) and PD-L1 (atezolizumab, avelumab) agents present a higher risk of myocarditis and acute myocardial infarction. Moreover, combination regimens involving PD-1/PD-L1 further elevate the risk of cardiac toxicity, underscoring the necessity for vigilant monitoring in patients with underlying heart disease.

**Clinical Trial Registration:**

https://www.crd.york.ac.uk/PROSPERO/, identifier CRD420261357478.

## 1 Introduction

Immune checkpoint inhibitors (ICIs), which enhance the human immune system to target tumors, have become one of the most effective therapeutic strategies in advanced cancer and have emerged as a cornerstone of standard cancer care (1). Therapeutic agents include PD-1 inhibitors (nivolumab, pembrolizumab, cemiplimab), PD-L1 inhibitors (atezolizumab, durvalumab, avelumab), CTLA-4 inhibitors (ipilimumab, tremelimumab), and the LAG-3 inhibitor (relatlimab), while numerous studies have investigated the efficacy and safety of ICIs, alone or combined with other agents, particularly in advanced or recurrent cancer (2). Not all patients achieve equivalent benefits from ICIs, and many experience immune-related adverse events (irAEs).

Because ICIs modulate autoreactivity, they have been associated with disinhibited cytotoxic T cells that may damage healthy tissues across multiple organs, leading to irAEs. Indeed, over 60%–80% of patients experience irAEs such as endocrinopathies, hepatitis, colitis, inflammatory arthritis, and pneumonitis (3, 4). Increasingly, irAEs related to cardiac toxicity have been reported during ICI treatments, including myocarditis, pericarditis, pericardial effusion, atherosclerotic deterioration, acute myocardial infarction, and heart failure. Different ICI types and agents cause varying types and incidences of cardiac toxicity (5, 6). Clinical manifestations range from asymptomatic or mild to severe symptoms such as chest pain and cardiogenic shock (7, 8). Severe presentations indicate poor prognosis, with mortality rates of 25% to 50% (9), often associated with combination therapies involving ICIs (10).

Accordingly, our study aims to identify ICI types and specific agents prone to cardiac toxicity, as well as combination therapy regimens most likely to induce cardiotoxic events. Although prior studies included numerous original articles (11–15), none applied network meta-analysis to rank ICI types and agents by cardiac toxicity risk nor utilized network analysis combined with pharmacovigilance of the FDA Adverse Event Reporting System (FAERS) database.

## 2 Methods

This research was conducted following the Preferred Reporting Items for Systematic Reviews and Meta-Analyses (PRISMA) guidelines, with the prospective protocol (CRD42020261357478) registered in an open repository (16, 17). Ethical approval was waived as no individual-level data were collected.

### 2.1 Study design and selection criteria

To comprehensively identify the ICIs most associated with cardiac toxicity, we conducted a systematic review and network meta-analysis. Eligible studies were randomized controlled trials (RCTs) involving adults (≥18 years) with any primary cancer diagnosis at any stage. Interventions included at least one ICI agent—PD-1, PD-L1, CTLA-4, or LAG-3—administered alone or in combination. Non-tumor research, only conference abstracts, studies without cardiac toxicity data (double-zero studies), and secondary analysis of previous studies were excluded. We searched PubMed, Embase, Cochrane Library, and the Clinical Trials Registry Platform (ClinicalTrials.gov) from inception to 30 September 2025, using the search terms detailed in **Supplementary Table 1**, without language or other restrictions. Literature update search was completed on 10 November 2025, with literature de-duplication and screening conducted in EndNote X8 software. Titles were screened independently in duplicate by two authors (CH and SYQ), followed by abstract and full-text review. Discrepancies were resolved via consensus or adjudicated by a third reviewer (ZQC).

### 2.2 Data extraction and outcomes

Two authors (CH and SYQ) independently extracted data, as presented in **Supplementary Table 2**, including the first author, publication year, cancer stage, clinical trial number, sample size, ICI type, agent, and dosage. Risk of bias was assessed independently by two authors (CH and SYQ) using the Cochrane Risk of Bias tool version 2 (RoB 2) (18), with discrepancies resolved by a third reviewer (ZQC).

The predefined primary outcomes were cardiac toxicity events attributed to ICIs, including incident rates of acute myocardial infarction, acute coronary syndrome, angina pectoris, heart failure, cardiac arrest, sudden cardiac death, atrial fibrillation, sinus tachycardia, ventricular tachycardia, conduction block, bradyarrhythmia, myocarditis, and pericardial effusion. These outcomes represent key manifestations of cardiac toxicity and were preplanned for subgroup analyses among different interventions. The Grading of Recommendations Assessment, Development and Evaluation (GRADE) approach was employed to assess the certainty of evidence for all pairwise comparisons (19).

### 2.3 Data analysis of network meta-analysis

The incidence of cardiac toxicity associated with ICIs, treated as a dichotomous variable, was expressed as odds ratios (ORs) with 95% confidence intervals (CIs). We first performed random-effects pairwise meta-analyses, followed by Bayesian network meta-analyses using a random-effects model with a common heterogeneity parameter across comparisons (20, 21). Statistical heterogeneity was quantified by the *I*^2^ statistic, with *I*^2^ greater than 50% indicating substantial heterogeneity. Meta-regression evaluated the impact of stratification on pairwise outcomes. Publication bias was assessed using Begg’s and Egger’s tests for different interventions to comprehensively evaluate pairwise meta-analysis results (22).

Network meta-analysis utilized a Bayesian multivariate framework. The Bayesian model follows a prior distribution, with 4 chains, 50,000 iterations, and 20,000 burn-in periods. The convergence diagnosis is visualized using a trace plot, and the fit index is determined by the deviation information criterion (DIC) value. The smaller the DIC value, the better. Surface under the cumulative ranking curve (SUCRA) scores (ranging from 0 to 1) ranked interventions from least to most likely to induce cardiac toxicity (23). Pairwise meta-analysis supplemented network meta-analysis, directly and indirectly comparing cardiac toxicity proportions among interventions via ORs and 95% CIs to evaluate significance. We evaluated incoherence between direct and indirect evidence globally by using the method design-by-treatment interaction and locally by using the node-splitting approach (24). For global and local inconsistencies, a *p*-value greater than 0.05 indicates the absence of inconsistency. For comparisons with more than 10 studies, contour-enhanced network funnel plots assessed publication bias. Statistical analyses were performed using R (version 4.4.1) with meta, netmeta, and gemtc packages and STATA (SE 15.0).

### 2.4 Methods for pharmacovigilance study

We extracted adverse drug event (ADE) reports associated with cardiac toxicity related to ICIs from the FAERS database, covering the period from 2011 (Q1) to 2026 (Q1), which is based on the marketing timeline of ICI agents. Only the latest reports submitted to the FDA were selected, and CASEID and PRIMARYID were used as key filters to remove duplicate records. All ADEs were coded using preferred terms (PT) from the Medical Dictionary for Regulatory Activities (MedDRA, Version 28.0), within system organ classes (SOC) of cardiac disorders. The common and product names of cemiplimab, nivolumab, durvalumab, avelumab, pembrolizumab, and atezolizumab were applied for data extraction (see details in **Supplementary Table 2**), with only reports in which an ICI was listed as the primary suspect (PS) drug included.

Our study used disproportionality analysis as the primary signal detection approach, supported by four statistical algorithms: reporting odds ratio (ROR), proportional reporting ratio (PRR), information component (IC), and empirical Bayes geometric mean (EBGM). For the determination of positive signals, for ROR, a signal required at least three reports and a lower 95% confidence interval (CI) greater than 1; for PRR, it required at least three reports, a PRR value of 2 or higher, and a chi-squared statistic of at least 4; for IC, the lower bound of the 95% CI (denoted IC025) had to exceed 0; and for EBGM, the lower 95% CI (EBGM05) needed to be greater than 2. A drug–ADE combination was considered a positive signal only if it simultaneously met the predefined positive criteria for all four algorithms (25, 26). The incidence of rare adverse reactions is typically defined as ≥0.01% and <0.1%, while the incidence of common adverse reactions ranges from ≥1% to <10%. Therefore, we conducted statistics based on PT with an incidence exceeding 0.01% of the population for each ICI (27).

The interval between adverse event occurrence and ICI use was used to estimate time-to-onset (TTO) analysis. We used cumulative distribution curves to present the time-to-onset characteristics of the most significant ADR following treatment with different ICIs, based on data from FAERS at the PT level to assess whether the reporting pattern varied over time (28). Moreover, we used the Ω shrinkage to measure drug–drug interactions, which is the most conservative method among multiple algorithms (29). When at least one type of drug was recorded in the report, the patient was defined as having a co-medication history. Statistical analyses were performed using R version 4.4.1.

## 3 Results

**Figure 1:**
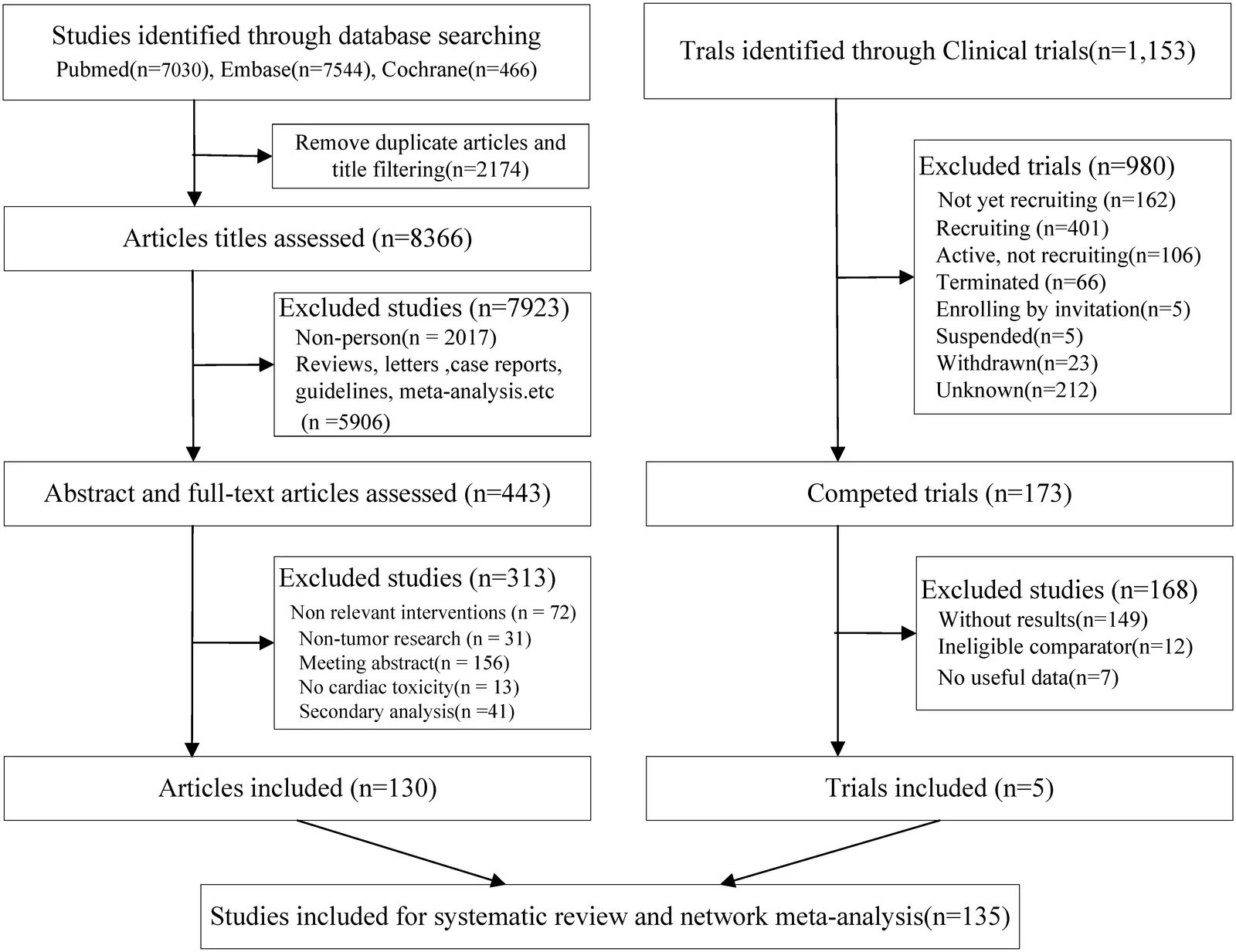
Diagram of the literature search and screening process.

Following our rigorous selection, 135 unique RCTs qualified for inclusion in the network meta-analysis and systematic review. Five RCTs were retrieved from ClinicalTrials.gov; the remaining 130 originated from published articles. The selection process is depicted in **Figure 1**, with reasons for exclusion and included study details provided. The RCTs spanned publication years 2010–2025. Baseline characteristics are summarized in **Table 1** and detailed in **Supplementary Table 3**. Studies with multiple comparisons were subdivided as substudies a, b, etc., and crossover studies were also divided into substudies, or only the outcomes of the first trial were taken. Quality assessment confirmed high methodological standards suitable for meta-analysis (**Supplementary Figure 1**).

**Table 1:** Summary of baseline characteristics.

Intervention type	Intervention agent	Control type	Control agent	Cancer type	No. of clinical trial
PD-1 + CTLA-4	Nivolumab + ipilimumab	PD-1	Nivolumab	CRC	NCT04008030[S1]
				NSCLC	NCT02785952[S19]
				Melanoma	NCT01844505[S23]
				Malignant pleural mesothelioma	NCT02716272[S58]
				Head and neck squamous cell carcinoma	NCT02823574[S109]
	Pembrolizumab + ipilimumab		Pembrolizumab	NSCLC	NCT03302234[S121]
		CTLA-4	Ipilimumab	Melanoma	NCT01927419[S22]
		SMTI	Sunitinib	RCC	NCT02231749[S40]
			Sunitinib/pazopanib	RCC	NCT02960906[S51]
		Chemotherapy	Pemetrexed + cisplatin	Malignant pleural mesothelioma	NCT02899299[S44]
		Placebo	–	SCLC	NCT02538666[S101]
PD-L1 + CTLA-4	Durvalumab + tremelimumab	PD-L1	Durvalumab	Endometrial cancer	NCT03015129[S59]
PD-L1 + CTLA-4 + chemotherapy	Durvalumab + tremelimumab + platinum-etoposide	PD-L1 + chemotherapy	Durvalumab + platinum-etoposide	SCLC	NCT03043872[S20]
	Durvalumab + tremelimumab + platinum-based		Durvalumab + platinum-based	NSCLC	NCT03164616[S26]
		Chemotherapy	Platinum-based	NSCLC	NCT03164616[S26]
PD-L1 + chemotherapy	Atezolizumab + bevacizumab + FOLFOXIRI	SMTI+chemotherapy	Bevacizumab + FOLFOXIRI	CRC	NCT03721653[S3]
	Atezolizumab + carboplatin + paclitaxel	Chemotherapy	Carboplatin + paclitaxel	Endometrial cancer	NCT03603184[S10]
	Atezolizumab + platinum-based		Platinum-based	NSCLC	NCT02486718[S16]
	Atezolizumab + carboplatin + nab-paclitaxel		Carboplatin + nab-paclitaxel	NSCLC	NCT02367781[S50]
	Atezolizumab + carboplatin/cisplatin + pemetrexed		Carboplatin/cisplatin + pemetrexed	NSCLC	NCT02657434[S66]
	Atezolizumab + lurbinectedin		Lurbinectedin	SCLC	NCT05091567[S70]
	Atezolizumab + trastuzumab		Trastuzumab	BC	NCT02924883[S81]
	Atezolizumab + nab-paclitaxel		Nab-paclitaxel	BC	NCT02425891[S98]
	Atezolizumab + carboplatin + etoposide		Carboplatin + etoposide	SCLC	NCT02763579[S107]
	Atezolizumab + paclitaxel		Paclitaxel	BC	NCT03125902[S119]
	Avelumab + carboplatin		Carboplatin	Epithelial ovarian cancer, fallopian tube cancer, or peritoneal cancer	NCT02718417[S38]
	Avelumab + carboplatin + paclitaxel		Carboplatin + paclitaxel	Endometrial cancer	NCT03503786[S69]
				Epithelial ovarian cancer, fallopian tube cancer, or peritoneal cancer	NCT02718417[S87]
	Durvalumab + azacitidine		Azacitidine	AML	NCT02775903[S43]
	Sugemalimab + platinum		Platinum	NSCLC	NCT03789604[S124]
PD-L1 + SMTI	Benmelstobart + anlotinib	SMTI	Sunitinib	RCC	NCT04523272[S42]
	Avelumab + axitinib			RCC	NCT02684006[S60]
	Atezolizumab + cobimetinib		Regorafenib	CRC	NCT02788279[S89]
	Atezolizumab + cobimetinib + vemurafenib		Cobimetinib + vemurafenib	Melanoma	NCT02908672[S112]
	Spartalizumab + dabrafenib + trametinib		Dabrafenib + trametinib	Melanoma	NCT02967692[S114]
PD-L1 + SMTI + chemotherapy	Atezolizumab + bevacizumab + carboplatin + pemetrexed	SMTI + chemotherapy	Bevacizumab + carboplatin + pemetrexed	Mesothelioma of pleura	NCT03762018[S17]
	Atezolizumab + bevacizumab + paclitaxel + carboplatin		Bevacizumab + paclitaxel + carboplatin	Epithelial ovarian cancer, fallopian tube cancer, or peritoneal cancer	NCT03038100[S39]
	Atezolizumab + bevacizumab + capecitabine		Bevacizumab + capecitabine	CRC	NCT02873195[S110]
	Atezolizumab + bevacizumab + platinum-based		Bevacizumab + platinum-based	Ovarian cancer	NCT02891824[S111]
	Avelumab + palbociclib + fulvestrant		Palbociclib + fulvestrant	BC	NCT03147287[S72]
		Chemotherapy	Fulvestrant	BC	NCT03147287[S72]
PD-1 + LAG-3 + chemotherapy	Nivolumab + BMS-986213 + XELOX	PD-1 + chemotherapy	Nivolumab + XELOX	Adenocarcinoma of the gastroesophageal junction	NCT03662659[S82]
PD-1 + radiotherapy	Durvalumab + radiotherapy	SMTI + radiotherapy	Cetuximab + radiotherapy	Head and neck squamous cell carcinoma	NCT03258554[S36]
PD-1 + chemotherapy	Nivolumab + platinum-based	Chemotherapy	Platinum-based	NSCLC	NCT02477826[S6]
	Nivolumab + carboplatin/paclitaxel/bevacizumab		Carboplatin/paclitaxel/bevacizumab	NSCLC	NCT03117049[S118]
	Pembrolizumab + paclitaxel + carboplatin		Paclitaxel + carboplatin	Endometrial cancer	NCT03914612[S14]
				BC	NCT03036488[S54]
	Pembrolizumab + carboplatin + paclitaxel/nab-paclitaxel		Carboplatin + paclitaxel/nab-paclitaxel	NSCLC	NCT02775435[S108]
	Pembrolizumab + pemetrexed + platinum		Pemetrexed + platinum	NSCLC	NCT02578680[S18]
				NSCLC	NCT03515837[S45]
	Pembrolizumab + cisplatin/carboplatin		Paclitaxel + cisplatin/carboplatin	Cervical cancer	NCT03635567[S27]
	Pembrolizumab + cisplatin		Cisplatin	Head and neck squamous cell carcinoma	NCT03040999[S33]
	Pembrolizumab + lenalidomide + dexamethasone		Lenalidomide + dexamethasone	Multiple myeloma	NCT02579863[S52]
	Pembrolizumab + cisplatin-based		Cisplatin-based	NSCLC	NCT03425643[S56]
	Pembrolizumab + fluorouracil/cisplatin/capecitabine/oxaliplatin		Fluorouracil/cisplatin/capecitabine/oxaliplatin	Adenocarcinoma of the stomach or gastroesophageal junction	NCT03675737[S62]
	Pembrolizumab + nab-paclitaxel/paclitaxel/gemcitabine + carboplatin		Nab-paclitaxel/paclitaxel/gemcitabine + carboplatin	BC	NCT02819518[S80]
	Pembrolizumab + cisplatin + fluoroura/capecitabine		Cisplatin + fluoroura/capecitabine	Gastric cancer	NCT02494583[S99]
	Pembrolizumab + etoposide + cisplatin/carboplatin		Placebo + etoposide + cisplatin/carboplatin	SCLC	NCT03066778[S117]
	Pembrolizumab + 5-fluorouracil + cisplatin/capecitabine + cisplatin		5-Fluorouracil + cisplatin/capecitabine + cisplatin	Advanced gastric or gastro-esophageal cancer	NCT03221426[S129]
	Tislelizumab + platinum-based		Platinum-based	NSCLC	NCT03663205[S31]
	Toripalimab + chemotherapy		–	Nasopharyngeal carcinoma	NCT03581786[S34]
			Paclitaxel + cisplatin	Esophageal squamous cancer	NCT03985670[S47]
	Tislelizumab + cisplatin/oxaliplatin + fluoropyrimidine		Cisplatin/oxaliplatin + fluoropyrimidine	Esophageal squamous cancer	NCT03783442[S46]
	Tislelizumab + carboplatin/cisplatin		Carboplatin/cisplatin	SCLC	NCT04005716[S134]
	Sintilimab + oxaliplatin/capecitabine		Oxaliplatin/capecitabine	CRC	NCT04304209[S48]
				CRC	ChiCTR2100052288[S53]
	Sintilimab + pemetrexed + platinum		Pemetrexed + platinum	NSCLC	NCT03607539[S122]
	Sintilimab + platinum + gemcitabine		Platinum + gemcitabine	NSCLC	NCT03629925[S123]
	Sintilimab + XELOX		XELOX	Gastric or gastroesophageal junction cancer	NCT03745170[S131]
	Sintilimab + cisplatin + paclitaxel/cisplatin + 5-fluorouracil		Cisplatin + paclitaxel/cisplatin + 5-fluorouracil	Gastric or gastroesophageal junction cancer	NCT03748134[S132]
	Sintilimab + pemetrexed + cisplatin		Pemetrexed + cisplatin	NSCLC	NCT03802240[S133]
	Camrelizumab + nab-paclitaxel		Nab-paclitaxel	Gastric cancer	NCT04294784[S55]
	Camrelizumab + paclitaxel + cisplatin		Paclitaxel + cisplatin	Esophageal squamous cell carcinoma	NCT03691090[S130]
	Serplulimab + cisplatin + 5-fluorouracil		Cisplatin + 5-fluorouracil	Esophageal squamous cancer	NCT03958890[S126]
	Toripalimab + platinum-based		Platinum-based	NSCLC	NCT04158440[S135]
PD-1 + SMTI	Avelumab + axitinib	SMTI	Sunitinib	RCC	NCT02684006[S9]
	Pembrolizumab + axitinib			RCC	NCT02853331[S68]
	Pembrolizumab + dabrafenib + trametinib		Dabrafenib + trametinib	Dabrafenib + trametinib	NCT02130466[S75]
	Pembrolizumab + lenvatinib		Lenvatinib	HCC	NCT03713593[S84]
		Chemotherapy	Paclitaxel + carboplatin	Endometrial cancer	NCT03884101[S86]
	Nivolumab + sitravatinib	Chemotherapy	Docetaxel	SCLC	NCT03906071[S73]
	Nivolumab + cabozantinib	SMTI	Sunitinib	Epithelial ovarian cancer, fallopian tube cancer, or peritoneal cancer	NCT02718417[S87]
				RCC	NCT03141177[S88]
	Pembrolizumab + ramucirumab	Usual care	–	NSCLC	NCT03971474[S63]
PD-1 + IDOi	Pembrolizumab + epacadostat	PD-1	Pembrolizumab	Melanoma	NCT02752074[85]
PD-1 + SMTI + chemotherapy	Pembrolizumab + trastuzumab + 5-fluorouracil + cisplatin/capecitabine + oxaliplatin	SMTI + chemotherapy	Trastuzumab + 5-fluorouracil + cisplatin/capecitabine + oxaliplatin	ER2-positive gastric cancer	NCT03615326[S25]
	Nivolumab + bevacizumab + mFOLFOX6		Bevacizumab + mFOLFOX6	CRC	NCT03414983[S30]
PD-1 + chemotherapy + radiotherapy	Nivolumab + temozolomide + radiotherapy	Chemotherapy + radiotherapy	Temozolomide + radiotherapy	Glioblastoma	NCT02667587[S103]
PD-1 + radiotherapy	Pembrolizumab + radium-223	Radiotherapy	Radium-223	PC	NCT03093428[S8]
PD-L1	Durvalumab	Placebo	–	NSCLC	NCT02125461[S2]
				Cervical cancer	NCT03830866[S125]
	Avelumab	Chemotherapy	Docetaxel	NSCLC	NCT02395172[S4]
		Placebo	–	Head and neck squamous cell carcinoma	NCT02952586[S113]
	Atezolizumab	Usual care	–	Bladder cancer	NCT02450331[S5]
				NSCLC	NCT02486718[S41]
		Chemotherapy	Docetaxel	NSCLC	NCT01903993[S15]
		Placebo	–	RCC	NCT03024996[S115]
PD-1	Pembrolizumab	Bifunctional fusion protein	Bintrafusp alfa	NSCLC	NCT03631706[S7]
		Chemotherapy	mFOLFOX6/FOLFIRI/bevacizumab	CRC	NCT02563002[S11]
			Docetaxel	NSCLC	NCT01905657[S21]
			Paclitaxel + cisplatin/carboplatin	Esophagus cancer	NCT02564263[S28]
			Carboplatin + pemetrexed	NSCLC	NCT02039674[S29]
			Platinum-based	NSCLC	NCT02220894[S37]
			Capecitabine/eribulin/gemcitabine/vinorelbine	BC	NCT02555657[S49]
			Methotrexate/docetaxel/cetuximab	Head and neck squamous cell carcinoma	NCT02252042[S79]
			Cisplatin + fluoroura/capecitabine	Gastric cancer	NCT02494583[S99]
		Placebo	–	Melanoma	NCT02362594[S13]
					NCT03553836[S32]
				NSCLC	NCT02504372[S65]
				RCC	NCT03142334[S67]
				Urothelial cancer	NCT02500121[S100]
				HCC	NCT03062358[S116]
				Esophagus cancer	NCT03189719[S120]
		Usual care	–	HCC	NCT02702401[S104]
	Camrelizumab	Chemotherapy	Docetaxel/irinotecan	Esophageal squamous cell carcinoma	NCT03099382[S24]
	Nivolumab	Chemotherapy	Pemetrexed + cisplatin	Malignant pleural mesothelioma	NCT02899299[S44]
			Everolimus	RCC	NCT01668784[S78]
			Paclitaxel/docetaxel	Esophageal squamous cell carcinoma	NCT02569242[S83]
		Placebo	–	Gastric cancer	NCT02267343[S97]
					NCT02746796[S106]
				Urothelial Cancer	NCT02632409[S102]
				Esophageal cancer or gastroesophageal junction cancer	NCT02743494[S105]
				Melanoma	NCT04099251[S128]
		Usual care	–	RCC	NCT03055013[74]
				NSCLC	NCT04025879[S127]
	Cemiplimab		Cemiplimab/platinum	NSCLC	NCT03088540[S57]
			Platinum-based	NSCLC	NCT03088540[S64]
CTLA-4	Ipilimumab	Placebo	–	Melanoma	NCT00636168[S12]
				PC	NCT01057810[S95]
		Usual care	–	Gastric cancer or gastric esophageal junction cancer	NCT01585987[S76]
		PD-1	Pembrolizumab	Melanoma	NCT01866319[S71]
	Tremelimumab	Placebo	–	Malignant mesothelioma	NCT01843374[S35]
		Usual care	–	Melanoma	NCT00257205[S61]
CTLA-4 + chemotherapy	Ipilimumab + dacarbazine	Chemotherapy	Dacarbazine	Melanoma	NCT00324155[S91]
	Ipilimumab + paclitaxel + carboplatin		Paclitaxel + carboplatin	SCLC	NCT00527735[S92]
				NSCLC	NCT00527735[S93]
				NSCLC	NCT01285609[S96]
CTLA-4 + radiotherapy	Ipilimumab + radiotherapy	Radiotherapy	–	PC	NCT00861614[S94]
CTLA4 + GP100	Ipilimumab + glycoprotein 100	CTLA4	Ipilimumab	Melanoma	NCT00094653[S90]
		GP100	Glycoprotein 100	Melanoma	NCT00094653[S90]

### 3.1 Pairwise meta-analysis on the incidence of all types of cardiac toxicity

The main outcome encompassed all types of cardiac toxicity arising from combined or standalone ICI therapy, including incidence rates of acute myocardial infarction, acute coronary syndrome, angina pectoris, heart failure, cardiac arrest, sudden cardiac death, atrial fibrillation, sinus tachycardia, ventricular tachycardia, conduction block, bradyarrhythmia, myocarditis, and pericardial effusion.

Pairwise meta-analysis revealed that, overall, combined or monotherapy with ICIs increased the incidence of acute myocardial infarction (OR: 1.537, 95% CI: 1.168–2.024; 87 studies). Significant differences were noted in PD-1  +  small molecule targeted inhibitors (SMTI) versus chemotherapy and PD-L1  + SMTI versus SMTI subgroups, without substantial heterogeneity (**Table 2**). Similarly, cardiac arrest incidence increased (OR: 1.509, 95% CI: 1.092–2.086; 64 studies), significantly observed in PD-1 versus chemotherapy groups. ICI monotherapy or combination also raised ventricular tachycardia incidence (OR: 1.510, 95% CI: 1.037–2.200; 41 studies), notably in PD-1 + CTLA-4 versus PD-1 comparisons. Furthermore, myocarditis risk increased (OR: 1.749, 95% CI: 1.237–2.472), with a significant difference in PD-1 versus the placebo subgroup (**Table 2**).

**Table 2:** Pairwise meta-analysis outcomes of Cardiotoxicity induced by immune checkpoint inhibitor according by different comparison.

Indicator	Comparison (no. of studies)	OR (95% CI)	*p, I* ^2^	*p*-value from meta-regression	Publication bias (Begg’s, Egger’s)	Grade
Acute myocardial infarction	Overall (*n* = 87)	1.537 (1.168, 2.024)^a^	0.997, 0.0%	0.780	0.308, 0.867	High
	CTLA-4 vs. placebo (*n* = 3)	1.871 (0.504, 6.942)	0.710, 0.0%		0.602, 0.226	Moderate
	CTLA-4 + chemotherapy vs. chemotherapy (*n* = 3)	1.448 (0.291, 7.205)	0.490, 0.0%		0.602, 0.168	Moderate
	PD-1 vs. chemotherapy (*n* = 10)	1.651 (0.750, 3.633)	0.728, 0.0%		0.421, 0.342	High
	PD-1 vs. placebo (*n* = 7)	1.196 (0.527, 2.714)	0.932, 0.0%		0.293, 0.062	High
	PD-1 vs. usual care (*n* = 4)	2.483 (0.582, 10.598)	0.997, 0.0%		0.174, 0.043^b^	Low
	PD-1 + SMTI vs. chemotherapy (*n* = 2)	7.841 (1.424, 43.179)^a^	0.880, 0.0%		0.317, –	Moderate
	PD-1 + chemotherapy vs. chemotherapy (*n* = 13)	1.273 (0.615, 2.635)	0.893, 0.0%		0.903, 0.657	High
	PD-1 + CTLA-4 vs. PD-1 (*n* = 5)	2.153 (0.428, 10.830)	0.149, 40.9%		0.327, 0.194	High
	PD-L1 vs. chemotherapy (*n* = 2)	0.382 (0.089,1.651)	0.917, 0.0%		0.317, –	Moderate
	PD-L1 vs. placebo (*n* = 5)	1.945 (0.471, 8.038)	0.781, 0.0%		0.624, 0.423	High
	PD-L1 vs. usual care (*n* = 2)	1.596 (0.196, 13.003)	0.608, 0.0%		0.317, –	Moderate
	PD-L1 + chemotherapy vs. chemotherapy (*n* = 11)	0.996 (0.422, 2.352)	0.813, 0.0%		0.312, 0.638	High
	PD-L1 + CTLA-4 + chemotherapy vs. chemotherapy (*n* = 2)	1.000 (0.104, 9.645)	0.339, 0.0%		0.317, –	Moderate
	PD-L1 + CTLA-4 + chemotherapy vs. PD-L1 + chemotherapy (*n* = 2)	1.000 (0.104, 9.641)	0.341, 0.0%		0.317, –	Moderate
	PD-L1 + SMTI vs. SMTI (*n* = 7)	2.548 (1.087, 5.970)^a^	0.644, 0.0%		0.881, 0.760	High
Acute coronary syndrome	Overall (*n* = 25)	1.117 (0.615, 2.028)	0.830, 0.0%	0.166	0.002, 0.001^b^	Moderate
	PD-1 vs. chemotherapy (*n* = 2)	3.028 (0.475, 19.288)	0.998, 0.0%		0.317, –	Moderate
	PD-1 vs. placebo (*n* = 2)	3.511 (0.398, 30.989)	0.492, 0.0%		0.317, –	Moderate
	PD-1 + chemotherapy vs. chemotherapy (*n* = 6)	0.838 (0.234, 2.994)	0.818, 0.0%		0.091, 0.847	High
	PD-1 + CTLA-4 vs. PD-1 (*n* = 3)	1.445 (0.227, 9.194)	0.548, 0.0%		0.602, 0.999	Moderate
Angina pectoris	Overall (*n* = 32)	1.388 (0.813, 2.371)	0.980, 0.0%	0.433	0.372, 0.107	High
	CTLA-4 + chemotherapy vs. chemotherapy (*n* = 2)	0.913 (0.100, 8.317)	0.361, 0.0%		0.317, –	Moderate
	PD-1 vs. chemotherapy (*n* = 3)	4.133 (0.682, 25.043)	0.905, 0.0%		0.602, 0.013^b^	Low
	PD-1 vs. placebo (*n* = 3)	2.329 (0.379, 14.326)	0.828, 0.0%		0.602, 0.016^b^	Low
	PD-1 vs. usual care (*n* = 3)	3.623 (0.587, 22.348)	0.964, 0.0%		0.117, 0.006^b^	Low
	PD-1 + chemotherapy vs. chemotherapy (*n* = 7)	0.623 (0.192, 2.025)	0.539, 0.0%		0.652, 0.562	High
Heart failure	Overall (*n* = 83)	1.042 (0.789, 1.377)	1.000, 0.0%	0.225	0.688, 0.778	High
	CTLA-4 vs. placebo (*n* = 3)	1.936 (0.515, 7.273)	0.474, 0.0%		0.602, 0.073	Moderate
	CTLA-4 + chemotherapy vs. chemotherapy (*n* = 2)	0.806 (0.200, 3.247)	0.727, 0.0%		0.317, –	Moderate
	PD-1 vs. chemotherapy (*n* = 9)	0.970 (0.451, 2.087)	0.856, 0.0%		0.404, 0.988	High
	PD-1 vs. placebo (*n* = 7)	1.819 (0.615, 5.383)	0.952, 0.0%		0.881, 0.112	High
	PD-1 vs. SMTI (*n* = 2)	1.120 (0.053, 23.650)	0.169, 47.2%		0.317, –	Moderate
	PD-1 + chemotherapy vs. chemotherapy (*n* = 14)	1.759 (0.847, 3.654)	0.993, 0.0%		0.139, 0.394	High
	PD-1 + CTLA-4 vs. PD-1 (*n* = 6)	1.433 (0.434, 4.733)	0.636, 0.0%		0.851, 0.479	High
	PD-1 + CTLA-4 vs. SMTI (*n* = 3)	0.317 (0.084, 1.199)	0.833, 0.0%		0.117, 0.461	Moderate
	PD-L1 vs. chemotherapy (*n* = 3)	1.530 (0.301, 7.775)	0.450, 0.0%		0.602, 0.219	Moderate
	PD-L1 vs. placebo (*n* = 2)	0.920 (0.139, 6.070)	0.411, 0.0%		0.317, –	Moderate
	PD-L1 vs. usual care (*n* = 2)	1.336 (0.250, 7.136)	0.569, 0.0%		0.317, –	Moderate
	PD-L1 + chemotherapy vs. chemotherapy (*n* = 10)	0.465 (0.181, 1.193)	0.976, 0.0%		0.853,0.467	High
	PD-L1 + CTLA-4 + chemotherapy vs. PD-L1 + chemotherapy (*n* = 2)	1.863 (0.073, 47.798)	0.134, 55.4%^c^		0.317, –	Low
	PD-L1 + SMTI vs. SMTI (*n* = 5)	1.194 (0.409, 3.486)	0.557, 0.0%		0.327, 0.560	High
Cardiac arrest	Overall (*n* = 64)	1.509 (1.092, 2.086)^a^	1.000, 0.0%	0.877	0.119, 0.664	High
	CTLA-4 vs. placebo (*n* = 3)	3.294 (0.566, 19.170)	0.958, 0.0%		0.602, 0.576	Moderate
	CTLA-4 + chemotherapy vs. chemotherapy (*n* = 2)	1.734 (0.551, 5.456)	0.814, 0.0%		0.317, –	Moderate
	PD-1 vs. chemotherapy (*n* = 6)	3.256 (1.028, 10.316)^a^	0.947, 0.0%		0.573, 0.637	High
	PD-1 vs. placebo (*n* = 5)	1.602 (0.511, 5.020)	0.918, 0.0%		0.624, 0.684	High
	PD-1 vs. usual care (*n* = 4)	1.051 (0.254, 4.344)	0.849, 0.0%		0.042, 0.056	Moderate
	PD-1 + chemotherapy vs. chemotherapy (*n* = 11)	1.269 (0.607, 2.653)	0.902, 0.0%		0.938, 0.552	High
	PD-1 + CTLA-4 vs. PD-1 (*n* = 4)	0.636 (0.148, 2.721)	0.595, 0.0%		0.497, 0.914	Moderate
	PD-1 + CTLA-4 vs. SMTI (*n* = 2)	0.897 (0.104, 7.724)	0.194, 40.8%		0.317, –	Moderate
	PD-L1 vs. placebo (*n* = 2)	0.669 (0.098, 4.578)	0.589, 0.0%		0.317, –	Moderate
	PD-L1 vs. usual care (*n* = 2)	2.076 (0.267, 16.119)	0.444, 0.0%		0.317, –	Moderate
	PD-L1 + chemotherapy vs. chemotherapy (*n* = 4)	3.491 (0.729, 16.710)	0.994, 0.0%		0.174, 0.000^b^	Moderate
	PD-L1 + SMTI vs. SMTI (*n* = 3)	1.002 (0.173, 5.800)	0.638, 0.0%		0.602, 0.999	Moderate
	PD-L1 + SMTI + chemotherapy vs. SMTI + chemotherapy (*n* = 2)	3.052 (0.315, 29.561)	0.537, 0.0%		0.317, –	Moderate
Sudden cardiac death	Overall (*n* = 8)	2.447 (0.795, 7.538)	0.971, 0.0%	0.739	0.386, 0.370	High
Atrial fibrillation	Overall (*n* = 69)	1.146 (0.856, 1.535)	0.995, 0.0%	0.751	0.399, 0.192	High
	CTLA-4 vs. PD-1 (*n* = 2)	0.333 (0.035, 3.214)	1.000,-		1.000, –	Moderate
	CTLA-4 vs. placebo (*n* = 3)	1.901 (0.686, 5.269)	0.551, 0.0%		0.602, 0.209	Moderate
	CTLA-4 + chemotherapy vs. chemotherapy (*n* = 3)	0.744 (0.271, 2.041)	0.576, 0.0%		0.602, 0.198	Moderate
	PD-1 vs. chemotherapy (*n* = 4)	1.228 (0.417,3.614)	0.720, 0.0%		0.174, 0.397	Moderate
	PD-1 vs. placebo (*n* = 5)	1.372 (0.451, 4.176)	0.697, 0.0%		1.000, 0.992	High
	PD-1 vs. usual care (*n* = 4)	0.578 (0.151, 2.214)	0.379, 2.8%		0.174, 0.022^b^	Moderate
	PD-1 + chemotherapy vs. chemotherapy (*n* = 11)	1.300 (0.666, 2.538)	0.968, 0.0%		0.312, 0.696	High
	PD-1 + CTLA-4 vs. CTLA-4 (*n* = 2)	2.046 (0.116, 36.026)	0.156, 50.3%^c^		0.317, –	Low
	PD-1 + CTLA-4 vs. PD-1 (*n* = 4)	1.618 (0.402, 6.513)	0.474, 0.0%		0.174, 0.231	Moderate
	PD-1 + SMTI vs. SMTI (*n* = 3)	1.827 (0.197, 16.982)	0.143,48.6%		0.602, 0.827	Moderate
	PD-L1 vs. chemotherapy (*n* = 2)	0.562 (0.118, 2.689)	0.707, 0.0%		0.317, –	Moderate
	PD-L1 vs. placebo (*n* = 2)	4.994 (0.582, 42.841)	0.999, 0.0%		0.317, –	Moderate
	PD-L1 + chemotherapy vs. chemotherapy (*n* = 7)	1.076 (0.375, 3.089)	0.593, 0.0%		0.072, 0.227	High
	PD-L1 + SMTI vs. SMTI (*n* = 3)	0.750 (0.209, 2.690)	0.596, 0.0%		0.602, 0.007	Moderate
Sinus tachycardia	Overall (*n* = 38)	1.310 (0.923, 1.860)	0.985, 0.0%	0.702	0.451, 0.155	High
	CTLA-4 vs. placebo (*n* = 2)	0.712 (0.074, 6.858)	0.512, 0.0%		0.317, –	Moderate
	CTLA-4 + chemotherapy vs. chemotherapy (*n* = 4)	0.598 (0.103, 3.470)	0.165, 41.2%		0.497, 0.744	Moderate
	PD-1 vs. chemotherapy (*n* = 4)	1.678 (0.703, 4.006)	0.505, 0.0%		0.497, 0.307	Moderate
	PD-1 vs. placebo (*n* = 2)	0.771 (0.080, 7.429)	0.492, 0.0%		0.317, –	Moderate
	PD-1 + chemotherapy vs. chemotherapy (*n* = 5)	1.813 (0.954, 3.445)	0.688, 0.0%		0.624, 0.495	High
	PD-1 + CTLA-4 vs. PD-1 (*n* = 2)	2.250 (0.496, 10.210)	0.837, 0.0%		0.317, –	Moderate
	PD-L1 + chemotherapy vs. chemotherapy (*n* = 2)	3.021 (0.314, 29.111)	0.997, 0.0%		0.317, –	Moderate
	PD-L1 + SMTI vs. SMTI (*n* = 3)	0.516 (0.089, 2.987)	0.834, 0.0%		0.602, 0.007^b^	Moderate
Ventricular tachycardia	Overall (*n* = 41)	1.510 (1.037, 2.200)^a^	0.974, 0.0%	0.346	0.016, 0.015^b^	High
	CTLA-4 + chemotherapy vs. chemotherapy (*n* = 3)	0.812 (0.140, 4.709)	0.478, 0.0%		0.602, 0.876	Moderate
	PD-1 vs. chemotherapy (*n* = 5)	0.840 (0.222, 3.187)	0.731, 0.0%		0.142, 0.284	High
	PD-1 vs. placebo (*n* = 3)	1.131 (0.178, 7.191)	0.626, 0.0%		0.602, 0.725	Moderate
	PD-1 vs. usual care (*n* = 2)	0.700 (0.073, 6.754)	0.524, 0.0%		0.317, –	Moderate
	PD-1 + chemotherapy vs. chemotherapy (*n* = 8)	1.340 (0.552, 3.255)	0.478, 0.0%		0.805, 0.058	High
	PD-1 + CTLA-4 vs. CTLA-4 (*n* = 2)	1.630 (0.746, 3.562)	0.961, 0.0%		0.317, –	Moderate
	PD-1 + CTLA-4 vs. PD-1 (*n* = 2)	4.052 (1.423, 11.541)^a^	0.858, 0.0%		0.317, –	Moderate
	PD-L1 vs. placebo (*n* = 2)	2.989 (0.310,28.794)	0.999, 0.0%		0.317, –	Moderate
	PD-L1 + chemotherapy vs. chemotherapy (*n* = 5)	0.785 (0.211, 2.928)	0.736, 0.0%		0.142, 0.880	High
Conduction block	Overall (*n* = 15)	1.644 (0.763, 3.542)	0.977, 0.0%	0.926	0.198, 0.587	High
	PD-1 vs. placebo (*n* = 3)	2.947 (0.580, 14.971)	0.890, 0.0%		0.602, 0.448	Moderate
	PD-1 + CTLA-4 vs. PD-1 (*n* = 3)	1.454 (0.228, 9.258)	0.541, 0.0%		0.117, 0.625	Moderate
Bradyarrhythmia	Overall (*n* = 19)	1.410 (0.704, 2.823)	0.994, 0.0%	0.672	0.010, 0.228	High
	PD-1 vs. placebo (*n* = 2)	1.245 (0.153, 10.157)	0.813, 0.0%		0.317, –	Moderate
	PD-1 + chemotherapy vs. chemotherapy (*n* = 2)	4.414 (0.453, 43.013)	0.764, 0.0%		0.317, –	Moderate
	PD-L1 vs. chemotherapy (*n* = 2)	2.169 (0.225, 20.897)	0.747, 0.0%		0.317, –	Moderate
	PD-L1 + chemotherapy vs. chemotherapy (*n* = 2)	2.111 (0.219, 20.357)	0.777, 0.0%		0.317, –	Moderate
	PD-L1 + SMTI vs. SMTI (*n* = 3)	1.069 (0.205, 5.563)	0.656, 0.0%		0.117, 0.219	Moderate
Myocarditis	Overall (*n* = 73)	1.749 (1.237, 2.472)^a^	1.000, 0.0%	0.270	0.432, 0.077	High
	PD-1 vs. chemotherapy (*n* = 5)	1.898 (0.507, 7.108)	0.777, 0.0%		0.327, 0.022^b^	High
	PD-1 vs. placebo (*n* = 8)	3.975 (1.382, 11.435)^a^	0.945, 0.0%		0.322, 0.982	High
	PD-1 + SMTI vs. chemotherapy (*n* = 2)	4.018 (0.443, 36.460)	0.810, 0.0%		0.317, –	Moderate
	PD-1 + chemotherapy vs. chemotherapy (*n* = 22)	1.806 (0.947, 3.446)	0.972, 0.0%		0.096, 0.512	High
	PD-1 + CTLA-4 vs. PD-1 (*n* = 3)	3.242 (0.645, 16.303)	0.812, 0.0%		0.602, 0.558	Moderate
	PD-1 + CTLA-4 vs. SMTI (*n* = 2)	1.904 (0.196, 18.454)	0.697, 0.0%		0.317, –	Moderate
	PD-L1 vs. chemotherapy (*n* = 2)	0.999 (0.104, 9.624)	0.341, 0.0%		0.317, –	Moderate
	PD-L1 vs. placebo (*n* = 2)	1.073 (0.132, 8.746)	0.406, 0.0%		0.317, –	Moderate
	PD-L1 vs. usual care (*n* = 2)	3.867 (0.427, 35.062)	0.820, 0.0%		0.317, –	Moderate
	PD-L1 + chemotherapy vs. chemotherapy (*n* = 5)	1.750 (0.509, 6.008)	0.952, 0.0%		0.327, 0.051	High
	PD-L1 + CTLA-4 + chemotherapy vs. chemotherapy (*n* = 2)	3.011 (0.312, 29.030)	0.997, 0.0%		0.317, –	Moderate
	PD-L1 + CTLA-4 + chemotherapy vs. PD-L1 + chemotherapy (*n* = 2)	3.010 (0.312, 29.019)	1.000, 0.0%		0.317, –	Moderate
	PD-L1 + SMTI vs. SMTI (*n* = 3)	1.102 (0.241, 5.042)	0.372, 0.0%		0.117, 0.174	Moderate
	PD-L1 + SMTI + chemotherapy vs. SMTI + chemotherapy (*n* = 4)	1.632 (0.349, 7.630)	0.967, 0.0%		0.497, 0.196	Moderate
Pericardial effusion	Overall (*n* = 37)	1.065 (0.693, 1.639)	0.991, 0.0%	0.823	0.012, 0.994	High
	CTLA-4 + chemotherapy vs. chemotherapy (*n* = 2)	0.695 (0.072, 6.729)	0.527, 0.0%		0.317, –	Moderate
	PD-1 vs. usual care (*n* = 3)	0.630 (0.120, 3.298)	0.429, 0.0%		0.117, 0.155	Moderate
	PD-1 + SMTI vs. chemotherapy (*n* = 2)	3.063 (0.318, 29.522)	0.990, 0.0%		0.317, –	Moderate
	PD-1 + chemotherapy vs. chemotherapy (*n* = 10)	1.285 (0.476, 3.464)	0.929, 0.0%		0.128, 0.836	High
	PD-1 + CTLA-4 vs. PD-1 (*n* = 5)	0.768 (0.246, 2.402)	0.435, 0.0%		1.000, 0.635	High
	PD-L1 vs. chemotherapy (*n* = 2)	0.738 (0.138, 3.937)	0.563, 0.0%		0.317, –	Moderate
	PD-L1 + chemotherapy vs. chemotherapy (*n* = 2)	2.616 (0.354, 19.327)	0.331, 0.0%		0.317, –	Moderate

^a^ Significant differences.

^b^ Possible publication bias.

^c^ Substantial heterogeneity.

Overall, minimal heterogeneity was observed in outcomes and subgroups demonstrating significant differences, indicating robustness. Publication bias was infrequently detected. Most GRADE assessments rated the evidence as moderate to high. The head-to-head meta-analysis confirmed that both combined and monotherapy ICIs elevate acute myocardial infarction, cardiac arrest, ventricular tachycardia, and myocarditis risks (**Table 2**). However, the specific ICI types or combinations most likely to induce specific cardiac toxicity risks—acute myocardial infarction, cardiac arrest, ventricular tachycardia, and myocarditis—remain unclear, necessitating further network meta-analysis to identify regimens posing the highest risks for these four cardiac toxicities.

### 3.2 Network meta-analysis on the incidence of significantly different types of cardiac toxicity

**Figure 2:**
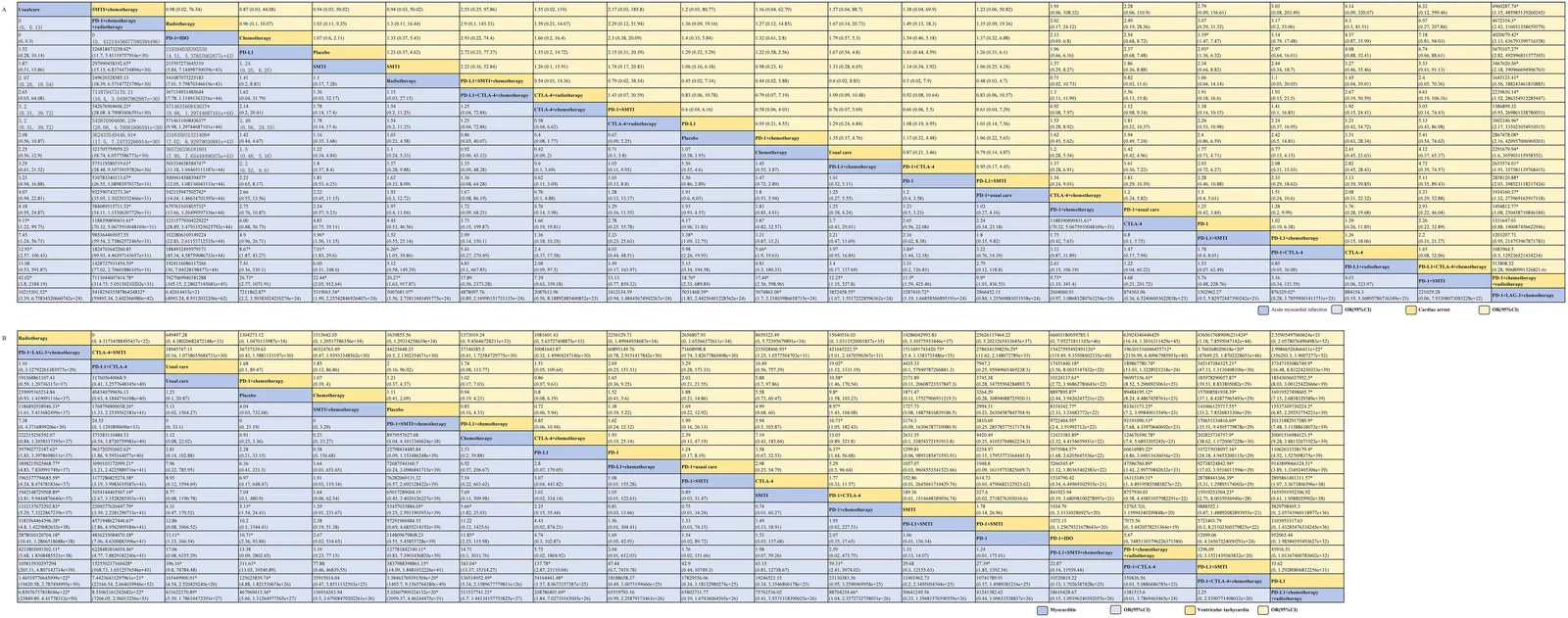
Comprehensive intervention type network meta-analysis of cardiac toxicity during the combination and separation application of immune checkpoint inhibitors with other overall interventions from four aspects: **(A)** acute myocardial infarction and cardiac arrest; **(B)** myocarditis and ventricular tachycardia. Odds ratios (ORs) are presented with 95% confidence intervals (CIs) in parentheses. Comparisons (row vs. column) should be read right to left and ordered by overall adverse event occurrence. The intervention in the bottom-right position ranks as the highest risk for cardiac toxicity per network meta-analysis of direct and indirect effects.

To identify combined or monotherapy ICI regimens most likely to cause acute myocardial infarction, cardiac arrest, ventricular tachycardia, and myocarditis, we performed an initial network meta-analysis. For acute myocardial infarction, the top 5 interventions with the highest SUCRA scores were PD-1 + LAG-3 + chemotherapy, PD-1 + SMTI, PD-L1 + radiotherapy, PD-1 + CTLA-4, and PD-L1 + SMTI (network plot in **Supplementary Figure 2**). Moreover, the global inconsistency of this outcome was relatively low (*p* = 0.9987, *I*^2^ = 11.34%), and there was no local inconsistency (**Supplementary Figure 3**). Significant differences frequently appeared between these top-ranked and lower-ranked interventions (**Figure 2A**), and publication bias was minimal (**Supplementary Figure 4**). For cardiac arrest, PD-1 + chemotherapy + radiotherapy, PD-L1 + CTLA-4 + chemotherapy, CTLA-4, PD-L1 + chemotherapy, and PD-1 ranked the highest, with PD-1 + chemotherapy + radiotherapy showing significant differences compared to others (**Figure 2A**). Regarding myocarditis, PD-L1 + chemotherapy + radiotherapy, PD-1 + CTLA-4 + chemotherapy, PD-1 + CTLA-4, PD-L1 + SMTI + chemotherapy, and PD-1 were top-ranked with significant differences (**Figure 2B**). The top interventions for ventricular tachycardia were PD-L1, PD-L1 + SMTI, PD-1 + chemotherapy + radiotherapy, PD-1 + IDO (immunometabolic regulators), and PD-1 + SMTI (**Figure 2B**), significantly differing from lower-ranked treatments. These findings collectively indicate that both PD-1 and PD-L1, as monotherapy or in combination, confer heightened cardiac toxicity risk. Consequently, we conducted a second network meta-analysis evaluating individual PD-1 and PD-L1 agents’ risks for these four cardiac toxicities.

**Figure 3:**
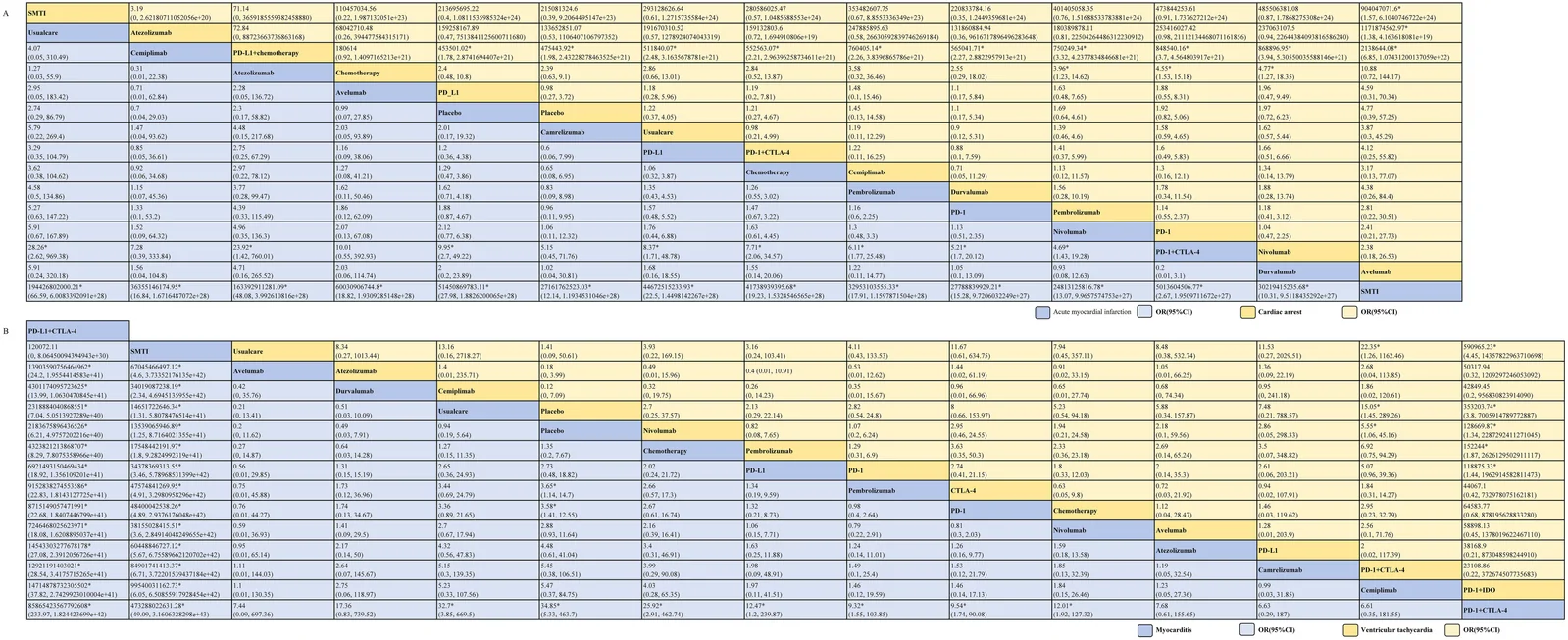
Comprehensive intervention type network meta-analysis of cardiac toxicity during the separation application of specific types of PD-1 and PD-L1 agents from four aspects: **(A)** acute myocardial infarction and cardiac arrest; **(B)** myocarditis and ventricular tachycardia. Odds ratios (ORs) are presented with 95% confidence intervals (CIs) in parentheses. Comparisons (row vs. column) should be read right to left and ordered by overall adverse event occurrence. The intervention in the bottom-right position ranks the highest for cardiac toxicity risk per network meta-analysis of direct and indirect effects.

To assess specific PD-1 and PD-L1 agents’ likelihood of causing acute myocardial infarction, cardiac arrest, myocarditis, and ventricular tachycardia, a second network meta-analysis was performed. For acute myocardial infarction, SMTI, durvalumab, PD-1 + CTLA-4, nivolumab, PD-1, and pembrolizumab ranked the highest, with significant differences among the top 3 (**Figure 3A**), with overall network plots and funnel plots shown in **Supplementary Figures 5**, **S7**. Moreover, the global inconsistency was relatively low (*p* = 0.9970, *I*^2^ = 8.07%), and there was no existence of local inconsistency (**Supplementary Figure 6**). For cardiac arrest risk, avelumab, nivolumab, PD-1, pembrolizumab, durvalumab, and cemiplimab ranked highly, with significant differences among the top 4 (**Figure 3A**). Myocarditis risk was the highest with PD-1 + CTLA-4, cemiplimab, camrelizumab, atezolizumab, nivolumab, and PD-1 monotherapy, with statistical significance among the top 2 pairs (**Figure 3B**). Ventricular tachycardia risk was elevated with PD-1 + IDO, PD-1 + CTLA-4, PD-L1, and avelumab, although significant differences were uncommon (**Figure 3B**). Overall, standalone PD-1 (cemiplimab, camrelizumab, nivolumab, pembrolizumab) and PD-L1 (atezolizumab, avelumab, durvalumab) agents are prone to causing these four cardiac toxicities. The potential additive risk from combination with CTLA-4 or SMTI remains unclear, warranting a third network meta-analysis.

**Figure 4:**
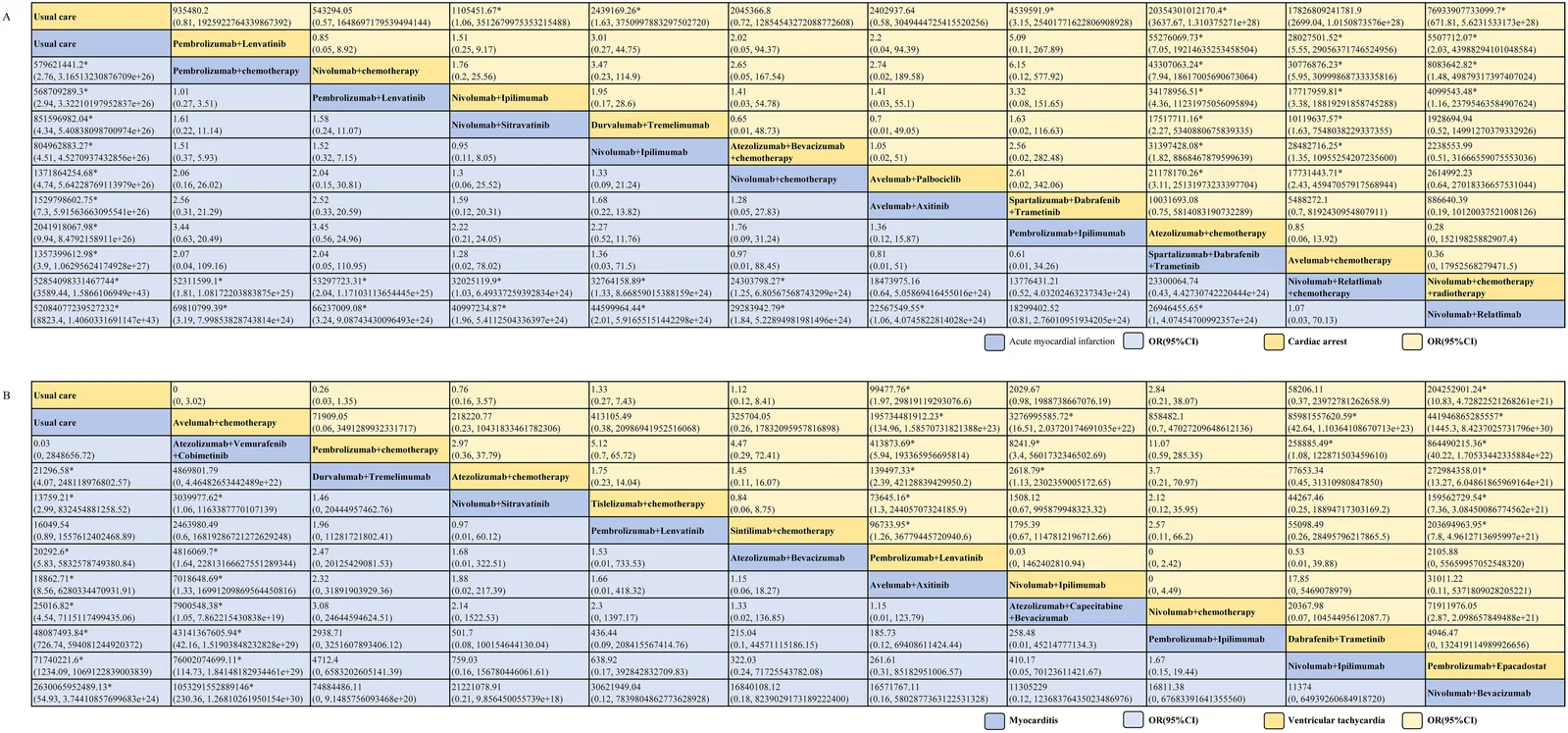
Comprehensive intervention type network meta-analysis of cardiac toxicity during the combination application of specific types of PD-1 and PD-L1 agents from four aspects: **(A)** acute myocardial infarction and cardiac arrest; **(B)** myocarditis and ventricular tachycardia. Odds ratios (ORs) are shown with 95% confidence intervals (CIs) in parentheses. Comparisons (row vs. column) should be read right to left and ordered by overall occurrence of adverse events. The intervention in the bottom-right position ranks the highest for cardiac toxicity risk based on network meta-analysis of direct and indirect effects.

To determine whether PD-1 (cemiplimab, camrelizumab, nivolumab, pembrolizumab) and PD-L1 (atezolizumab, avelumab, durvalumab) agents combined with CTLA-4 or SMTI exacerbate cardiac toxicity, a third network meta-analysis was conducted. For acute myocardial infarction, nivolumab + bevacizumab, nivolumab + ipilimumab, pembrolizumab + ipilimumab, atezolizumab + capecitabine + bevacizumab, and avelumab + axitinib ranked highly, with significant differences seen in the top 2 comparisons (**Figure 4A**). Network league charts displayed the top 10 interventions plus usual care; overall network plots and funnel plots are shown in **Supplementary Figures 8**, **S10**. Furthermore, the global inconsistency was relatively low (*p* = 1.0000, *I*^2^ = 6.76%), and there was no local inconsistency (**Supplementary Figure 9**). For cardiac arrest risk, pembrolizumab + epacadostat, dabrafenib + trametinib, nivolumab + chemotherapy, nivolumab + ipilimumab, and pembrolizumab + lenvatinib ranked the highest, with significant differences among the top 3 (**Figure 4A**). Regarding myocarditis risk, nivolumab + relatlimab, nivolumab + relatlimab + chemotherapy, spartalizumab + dabrafenib + trametinib, pembrolizumab + ipilimumab, and avelumab + axitinib ranked the highest, with significant differences in the top 2 comparisons (**Figure 4B**). For ventricular tachycardia, nivolumab + chemotherapy + radiotherapy, avelumab + chemotherapy, atezolizumab + chemotherapy, spartalizumab + dabrafenib + trametinib, and avelumab + palbociclib ranked the highest, with significant differences observed among the top 2 (**Figure 4B**). Overall, PD-1/PD-L1 combination therapy presents a higher cardiac toxicity risk, consistent with previous pairwise meta-analysis findings. Clinically, cardiac toxicity indicators should be monitored closely in patients receiving nivolumab, avelumab, pembrolizumab, and their combinations, especially in those with cardiovascular comorbidities, to enable individualized safety management.

### 3.3 Disproportionality analysis

Based on our three network meta-analyses identifying ICIs prone to adverse cardiac reactions, we conducted disproportionality analysis on agents available in the FAERS database. As camrelizumab is manufactured in China, its data are unavailable in FAERS; therefore, we analyzed PD-1 (cemiplimab, nivolumab, pembrolizumab) and PD-L1 (atezolizumab, avelumab, durvalumab) agents for ICI-related cardiac toxicity. The first step was to extract and compare the clinical characteristics of the six ICIs mentioned above and to determine that there was no significant difference in the proportion of the six ICIs in terms of gender, age, weight, reporter type, seriousness, fatal outcome, and outcome type, confirming baseline balance among the six ICIs for subsequent ADR comparisons (**Supplementary Table 4**).

The monotherapy disproportionality results are presented in **Table 3**. The study found that among the six ICIs mentioned above, the risk of myocarditis ranks the highest, and immune-mediated myocarditis is a broad concept that encompasses all myocardial injuries driven by immune responses, whereas autoimmune myocarditis is a narrow subset of immune-mediated myocarditis. Significant signals were detected in all ICIs mentioned above. These outcomes demonstrate that all ICIs mentioned above carry a risk of causing myocarditis, which is consistent with the results of our network meta-analysis. For acute coronary syndrome, avelumab and atezolizumab monotherapy showed a risk of causing this condition. All ICIs except avelumab showed a risk of causing pericardial effusion. Moreover, both nivolumab and atezolizumab posed a risk of causing pericarditis (**Table 3**).

**Table 3:** Positive PTs with ≥0.1% adverse event reports in SOCs of cardiac disorders of ICIs.

ICI type	PTs	Number of reports	ROR (95% Cl)	PRR (*χ*^2^)	EBGM (EBGM05)	IC (IC025)
Cemiplimab	Myocarditis*	58	**42.78 (33.01–55.44)**	**42.44 (2,332.5)**	**42.18 (32.55)**	**5.40 (4.26)**
	Cardiac failure*	18	**2.10 (1.32–3.33)**	**2.09 (10.28)**	**2.09 (1.32)**	**1.07 (0.32)**
	Cardiac arrest	14	**1.80 (1.06**–**3.03)**	1.79 (4.93)	1.79 (1.06)	0.84 (0.02)
	Atrial fibrillation	13	1.24 (0.72–2.13)	1.24 (0.59)	1.24 (0.72)	0.31 (−0.49)
	Cardiac disorder	8	0.85 (0.43–1.71)	0.86 (0.20)	0.86 (0.43)	−0.23 (−1.17)
	Immune-mediated myocarditis*	8	**38.63 (19.27**–**77.43)**	**38.59 (291.24)**	**38.37 (19.14)**	**5.26 (1.93)**
	Pericardial effusion*	7	**2.82 (1.34**–**5.92)**	**2.82 (8.21)**	**2.82 (1.34)**	**1.49 (0.18)**
	Myocardial infarction	7	0.49 (0.23–1.02)	0.49 (3.79)	0.49 (0.23)	−1.04 (−1.96)
	Tachycardia	6	0.65 (0.29–1.45)	0.65 (1.1)	0.65 (0.29)	−0.61 (−1.63)
	Bradycardia	6	1.1 (0.49–2.45)	1.10 (0.05)	1.10 (0.49)	0.14 (−0.97)
	Cardio-respiratory arrest	5	1.31 (0.54–3.14)	1.31 (0.36)	1.31 (0.54)	0.39 (−0.86)
	Arrhythmia	5	1.08 (0.45–2.61)	1.08 (0.03)	1.08 (0.45)	0.12 (−1.08)
	Acute myocardial infarction	5	1.74 (0.72–4.17)	1.74 (1.56)	1.74 (0.72)	0.80 (−0.55)
	Cardiac failure congestive	4	0.54 (0.20–1.44)	0.54 (1.57)	0.54 (0.20)	−0.89 (−2.04)
	Pericarditis	4	2.29 (0.86–6.11)	2.29 (2.91)	2.29 (0.86)	1.20 (−0.43)
	Cardiomyopathy	4	**2.86 (1.07**–**7.63)**	**2.86 (4.85)**	2.86 (1.07)	1.52 (−0.23)
	Autoimmune myocarditis*	3	**97.09 (31.05**–**303.56)**	**97.05 (281.12)**	**95.68 (30.60)**	**6.58 (0.5)**
	Palpitations	3	0.24 (0.08–0.75)	0.24 (7.14)	0.24 (0.08)	−2.05 (−3.19)
	Angina pectoris	3	1.13 (0.36–3.51)	1.13 (0.05)	1.13 (0.36)	0.18 (−1.31)
	Atrioventricular block	3	**3.87 (1.25**–**12.01)**	**3.87 (6.39)**	3.87 (1.25)	1.95 (−0.27)
	Acute coronary syndrome	3	**3.65 (1.18**–**11.32)**	**3.65 (5.76)**	3.64 (1.17)	1.87 (−0.31)
**Nivolumab**	Myocarditis*	705	**21.35 (19.77**–**23.06)**	**21.28 (1**,**2587.09)**	**19.73 (18.27)**	**4.30 (4.15)**
	Atrial fibrillation	466	**1.71 (1.56**–**1.88)**	1.71 (137.18)	1.71 (1.56)	0.77 (0.64)
	Cardiac failure	455	**2.05 (1.87**–**2.24)**	**2.04 (241.01)**	2.04 (1.86)	**1.03 (0.89)**
	Pericardial effusion*	353	**5.57 (5.02**–**6.20)**	**5.57 (1**,**294.65)**	**5.47 (4.92)**	**2.45 (2.28)**
	Immune-mediated myocarditis*	338	**82.03 (72.58**–**92.72)**	**81.88 (20**,**498.54)**	**62.39 (55.2)**	**5.96 (5.55)**
	Myocardial infarction	264	0.71 (0.63–0.80)	0.71 (32.28)	0.71 (0.63)	−0.50 (−0.68)
	Tachycardia	202	0.85 (0.74–0.97)	0.85 (5.64)	0.85 (0.74)	−0.24 (−0.44)
	Cardiac arrest	200	0.99 (0.86–1.13)	0.99 (0.04)	0.99 (0.86)	−0.02 (−0.22)
	Cardiac disorder	137	0.56 (0.48–0.66)	0.56 (46.56)	0.56 (0.48)	−0.83 (−1.07)
	Acute myocardial infarction	130	**1.74 (1.47**–**2.07)**	1.74 (40.83)	1.74 (1.46)	0.80 (0.54)
	Arrhythmia	124	1.04 (0.87–1.24)	1.04 (0.15)	1.04 (0.87)	0.05 (−0.21)
	Pericarditis*	121	**2.68 (2.24**–**3.21)**	**2.68 (126.51)**	**2.67 (2.23)**	**1.41 (1.13)**
	Cardiomyopathy*	117	**3.25 (2.71**–**3.90)**	**3.25 (179.97)**	**3.22 (2.68)**	**1.69 (1.39)**
	Cardio-respiratory arrest	100	1.01 (0.83–1.22)	1.01 (0.83)	1.01 (0.83)	0.01 (−0.28)
	Atrioventricular block complete*	92	**5.56 (4.53**–**6.84)**	**5.56 (337.06)**	**5.47 (4.45)**	**2.45 (2.08)**
	Cardiac tamponade*	90	**7.01 (5.68**–**8.64)**	**7.01 (451.21)**	**6.85 (5.55)**	**2.78 (2.38)**
	Cardiac failure congestive	89	0.46 (0.37–0.57)	0.46 (56.04)	0.46 (0.38)	−1.11 (−1.41)
	Palpitations	77	0.24 (0.19–0.30)	0.24 (187.58)	0.24 (0.19)	−2.06 (−2.38)
	Bradycardia	73	0.51 (0.41–0.65)	0.51 (33.46)	0.52 (0.41)	−0.96 (1.28)
	Sinus tachycardia	70	**2.03 (1.60**–**2.57)**	**2.03 (36.22)**	2.02 (1.60)	**1.01 (0.65)**
Durvalumab	Myocarditis*	129	**19.87 (16.70**–**23.65)**	**19.80 (2**,**271.38)**	**19.54 (16.42)**	**4.29 (3.84)**
	Cardiac failure	78	**1.89 (1.51**–**2.36)**	1.89 (32.69)	1.89 (1.51)	0.92 (0.58)
	Myocardial infarction	73	1.06 (0.84–1.33)	1.06 (0.23)	1.06 (0.84)	0.08 (−0.26)
	Immune-mediated myocarditis*	70	**73.71 (57.95**–**93.76)**	**73.56 (4**,**760.21)**	**69.94 (54.98)**	**6.13 (4.8)**
	Atrial fibrillation	65	**1.29 (1.01**–**1.64)**	1.29 (4.21)	1.29 (1.01)	0.37 (0)
	Pericardial effusion*	47	**3.95 (2.97**–**5.27)**	**3.95 (103.28)**	**3.94 (2.96)**	**1.98 (1.48)**
	Cardiac arrest	33	0.88 (0.63–1.24)	0.88 (0.53)	0.88 (0.63)	−0.18 (−0.67)
	Tachycardia	33	0.75 (0.53–1.05)	0.75 (2.79)	0.75 (0.53)	−0.42 (−0.90)
	Cardiac disorder	32	0.71 (0.50–1.01)	0.71 (3.72)	0.71 (0.50)	−0.49 (−0.98)
	Cardio-respiratory arrest	28	**1.53 (1.05**–**2.21)**	1.52 (5.05)	1.52 (1.05)	0.61 (0.05)
	Acute myocardial infarction	24	**1.74 (1.16**–**2.59)**	1.74 (7.49)	1.74 (1.16)	0.80 (0.18)
	Pericarditis	22	**2.63 (1.73**–**3.99)**	**2.63 (22.13)**	2.62 (1.73)	**1.39 (0.69)**
	Cardiomyopathy*	22	**3.29 (2.16**–**4.99)**	**3.28 (34.87)**	**3.28 (2.16)**	**1.71 (0.97)**
	Arrhythmia	20	0.90 (0.58–1.40)	0.90 (0.20)	0.90 (0.58)	−0.15 (−0.77)
	Cardiac tamponade*	17	**7.03 (4.37**–**11.33)**	**7.03 (87.51)**	**7.00 (4.35)**	**2.81 (1.71)**
	Cardiac failure congestive	16	0.45 (0.28–0.73)	0.45 (10.81)	0.45 (0.28)	−1.15 (−1.81)
Avelumab	Myocarditis*	27	**27.89 (19.10**–**40.73)**	**27.74 (694.19)**	**27.67 (18.94)**	**4.79 (3.28)**
	Tachycardia	16	**2.46 (1.51**–**4.02)**	**2.46 (13.82)**	2.46 (1.5)	**1.30 (0.48)**
	Cardiac failure	16	**2.63 (1.61**–**4.29)**	**2.62 (16.06)**	2.62 (1.60)	**1.39 (0.56)**
	Atrial fibrillation	14	**1.88 (1.11**–**3.18)**	1.88 (5.75)	1.88 (1.11)	**0.91 (0.08)**
	Cardiac arrest	12	**2.17 (1.23**–**3.82)**	**2.17 (7.55)**	2.17 (1.23)	**1.12 (0.19)**
	Myocardial infarction	10	0.98 (0.53**–**1.82)	0.98 (0.00)	0.98 (0.53)	−0.03 (−0.90)
	Bradycardia	8	**2.07 (1.03–4.14)**	**2.07 (4.41)**	2.07 (1.03)	1.05 (−0.08)
	Autoimmune myocarditis*	5	**230.36 (94.83-559.59)**	**230.13 (1**,**113.66)**	**224.7 (92.5)**	**7.81 (1.36)**
	Cardio-respiratory arrest	5	1.84 (0.77–4.43)	1.84 (1.92)	1.84 (0.77)	0.88 (−0.49)
	Cardiac disorder	5	0.75 (0.31–1.81)	0.75 (0.41)	0.75 (0.31)	−0.41 (−1.53)
	Palpitations	5	0.57 (0.24–1.36)	0.57 (1.64)	0.57 (0.24)	−0.82 (−1.89)
	Arrhythmia	5	1.53 (0.64–3.68)	1.53 (0.92)	1.53 (0.64)	0.61 (−0.69)
	Immune-mediated myocarditis*	5	**33.94 (14.1**–**81.72)**	**33.91 (159.14)**	**33.79 (14.04)**	**5.08 (1.20)**
	pericardial effusion	4	2.27 (0.85**–**6.05)	2.27 (2.84)	2.27 (0.85)	1.18 (−0.44)
	Acute coronary syndrome*	4	**6.86 (2.57–18.28)**	**6.85 (19.98)**	**6.85 (2.57)**	**2.78 (0.37)**
	Pericarditis	4	**3.23 (1.21–8.61)**	**3.23 (6.15)**	3.23 (1.21)	1.69 (−0.13)
	Cardiomyopathy	4	**4.04 (1.51–10.76)**	**4.03 (9.12)**	4.03 (1.51)	**2.01 (0.04)**
	Acute myocardial infarction	4	1.96 (0.73**–**5.22)	1.96 (1.87)	1.96 (0.73)	0.97 (−0.58)
	Atrioventricular block*	4	**7.28 (2.73–19.41)**	**7.28 (21.64)**	**7.27 (2.73)**	**2.86 (0.40)**
	Angina pectoris	4	2.13 (0.80**–**5.67)	2.13 (2.38)	2.13 (0.80)	1.09 (−0.50)
	Atrioventricular block complete*	3	**6.53 (2.11–20.27)**	**6.53 (14.04)**	**6.53 (2.10)**	**2.71 (0.01)**
	Ventricular fibrillation	3	**4.35 (1.40–13.49)**	**4.35 (7.73)**	4.35 (1.40)	2.12 (−0.20)
Pembrolizumab	Myocarditis*	644	**19.94 (18.40**–**21.60)**	**19.87 (10**,**739.13)**	**18.56 (17.13)**	**4.21 (4.06)**
	Immune-mediated myocarditis*	517	**155.46 (139.46**–**173.29)**	**155.01 (49**,**958.61)**	**49**,**958.61 (88.15)**	**6.62 (6.22)**
	Cardiac failure	466	**2.16 (1.97**–**2.37)**	**2.16 (286.82)**	2.15 (1.96)	**1.10 (0.96)**
	Atrial fibrillation	266	1.00 (0.89–1.13)	1.00 (0.00)	1.00 (0.89)	0.00 (−0.17)
	Pericardial effusion*	254	**4.10 (3.62**–**4.64)**	**4.10 (586.21)**	**4.05 (3.58)**	**2.02 (1.82)**
	Myocardial infarction	202	0.56 (0.48–0.64)	0.56 (71.66)	0.56 (0.48)	−0.84 (−1.04)
	Cardiac arrest	166	0.84 (0.72–0.98)	0.84 (4.89)	0.84 (0.72)	−0.25 (−0.47)
	Tachycardia	161	0.69 (0.59–0.81)	0.69 (21.70)	0.69 (0.60)	−0.53 (−0.75)
	Cardiac disorder	115	0.49 (0.40–0.58)	0.49 (62.46)	0.49 (0.41)	−1.04 (−1.30)
	Cardiomyopathy*	107	**3.06 (2.53**–**3.70)**	**3.06 (146.41)**	**3.03 (2.51)**	**1.60 (1.29)**
	Arrhythmia	103	0.89 (0.73–1.07)	0.89 (1.53)	0.89 (0.73)	−0.18 (−0.46)
	Cardiac tamponade*	98	**7.88 (6.44**–**9.63)**	**7.87 (571.25)**	**7.68 (6.28)**	**2.94 (2.55)**
	Palpitations	97	0.31 (0.25–0.38)	0.31 (149.78)	0.31 (0.25)	−1.69 (−1.97)
	Acute myocardial infarction	96	**1.32 (1.08**–**1.62)**	1.32 (7.50)	1.32 (1.08)	**0.40 (0.10)**
	Atrioventricular block complete*	95	**5.92 (4.83**–**7.26)**	**5.92 (379.87)**	**5.81 (4.74)**	**2.54 (2.17)**
	Cardio-respiratory arrest	87	0.90 (0.73–1.11)	0.90 (0.95)	0.90 (0.73)	−0.15 (−0.46)
	Cardiac failure congestive	76	0.40 (0.32–0.51)	0.41 (66.31)	0.41 (0.32)	−1.03 (−1.62)
	Pericarditis	76	**1.73 (1.38**–**2.17)**	1.73 (23.2)	1.72 (1.38)	**0.79 (0.44)**
	Cardiogenic shock	69	**1.75 (1.38**–**2.22)**	1.75 (22.18)	1.75 (1.38)	**0.81 (0.44)**
	Stress cardiomyopathy*	62	**3.49 (2.72**–**4.49)**	**3.49 (108.82)**	**3.46 (2.69)**	**1.79 (1.37)**
Atezolizumab	Myocarditis*	267	**21.68 (19.19**–**24.5)**	**21.60 (5**,**094.79)**	**21.00 (18.59)**	**4.39 (4.11)**
	Cardiac failure*	230	**2.91 (2.55**–**3.31)**	**2.90 (285.55)**	**2.89 (2.54)**	**1.53 (1.33)**
	Atrial fibrillation	147	1**.52 (1.29**–**1.78)**	1.51 (25.69)	1.51 (1.29)	**0.60 (0.36)**
	Myocardial infarction	134	1.01 (0.85–1.19)	1.01 (0.01)	1.01 (0.85)	0.01 (−0.24)
	Pericardial effusion*	110	**4.82 (4.00**–**5.82)**	**4.82 (330.49)**	**4.79 (3.97)**	**2.26 (1.94)**
	Cardiac arrest	96	**1.33 (1.09**–**1.63)**	1.33 (7.91)	1.33 (1.09)	**0.41 (0.11)**
	Acute myocardial infarction	66	**2.49 (1.95**–**3.17)**	**2.48 (58.33)**	2.48 (1.95)	**1.31 (0.92)**
	Pericarditis*	59	**3.67 (2.84**–**4.74)**	**3.67 (113.93)**	**3.65 (2.83)**	**1.87 (1.43)**
	Tachycardia	55	0.65 (0.50–0.84)	0.65 (10.55)	0.65 (0.50)	−0.63 (−1.00)
	Arrhythmia	44	1.03 (0.77–1.39)	1.03 (0.05)	1.03 (0.77)	0.05 (−0.39)
	Cardiac disorder	41	0.47 (0.35–0.64)	0.47 (23.99)	0.47 (0.35)	−1.08 (−1.50)
	Cardio-respiratory arrest	37	1.05 (0.76–1.44)	1.05 (0.07)	1.05 (0.76)	0.06 (−0.41)
	Cardiomyopathy*	36	**2.79 (2.01**–**3.88)**	**2.79 (41.29)**	**2.79 (2.01)**	**1.48 (0.93)**
	Cardiac failure congestive	34	0.50 (0.35–0.69)	0.50 (17.45)	0.50 (0.35)	−1.01 (−1.48)
	Angina pectoris	29	1.18 (0.82–1.70)	1.18 (0.82)	1.18 (0.82)	0.24 (−0.29)
	Immune-mediated myocarditis*	29	**15.35 (10.63**–**22.18)**	**15.34 (380.84)**	**15.05 (10.42)**	**3.91 (2.82)**
	Bradycardia	28	0.55 (0.38–0.80)	0.56 (9.98)	0.56 (0.38)	−0.85 (−1.36)
	Acute coronary syndrome*	28	**3.69 (2.55**–**5.36)**	**3.69 (54.73)**	**3.68 (2.54)**	**1.88 (1.21)**
	Atrial flutter*	27	**3.65 (2.50**–5**.32)**	**3.64 (51.55)**	**3.63 (2.49)**	**1.86 (1.18)**
	Atrioventricular block complete*	27	**4.53 (3.10**–**6.62)**	**4.53 (73.84)**	**4.51 (3.09)**	**2.17 (1.46)**
	Palpitations	26	0.23 (0.15–0.33)	0.23 (68.71)	0.23 (0.15)	−2.14 (−2.65)

Bold indicates a positive signal.

*Positive PT.

**Figure 5:**
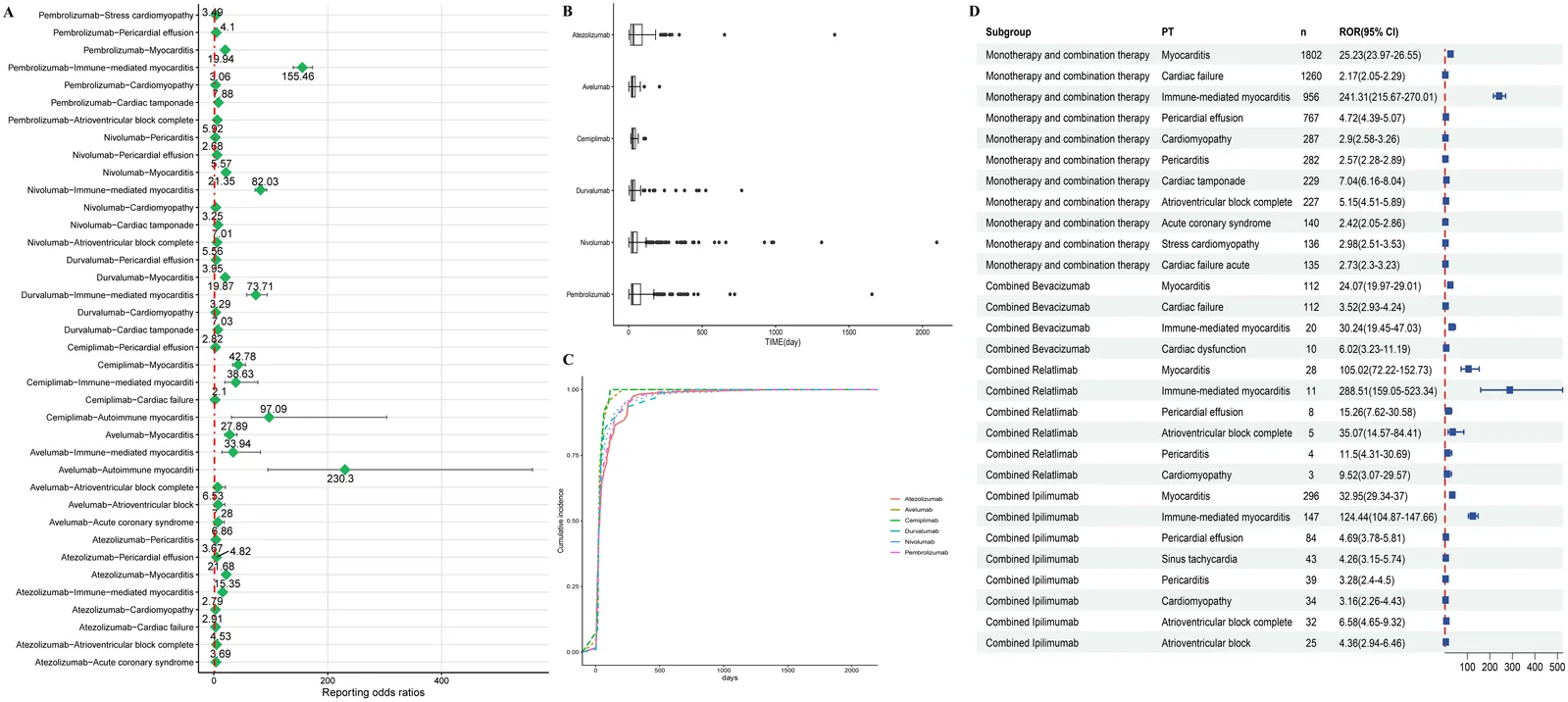
Signal values for ICI-associated cardiac toxicity based on the FAERS database. **(A)** Forest plot shows the ROR of ICI-related cardiac toxicity under different ICI monotherapy strategies. **(B)** Boxplot shows the time-to-onset of myocarditis of different ICI treatment. **(C)** Cumulative plot shows the time-to-onset of myocarditis of different ICI treatment. **(D)** Forest plot shows the ROR of ICI-related cardiac toxicity under different ICI combination therapy strategies.

We presented the PT with positive results for all four algorithms as ROR values in a forest plot and found that immune-mediated myocarditis had the highest value, indicating that it should be the ADE with the highest specificity for ICIs (**Figure 5A**). Subsequently, we conducted a TTO analysis of the three types of myocarditis; both box plots and cumulative plots demonstrated that myocarditis is most likely to occur during the early stages of medication. Monitoring should be carried out during the early stages of medication, especially within 30 days (**Figures 5B,C**). For the co-medication analysis, in addition to myocarditis as the main PT, we found that the combination of bevacizumab increased the risk of cardiac failure and cardiac dysfunction. Additionally, the combination of relatlimab significantly increased the risk of pericarditis, pericardial effusion, and cardiomyopathy. Moreover, the combination of ipilimumab increased the risk of atrioventricular block completed (**Figure 5D**). In summary, the disproportionality analysis based on the FAERS database further confirms that all six ICIs carry a risk of causing myocarditis, especially immune-mediated myocarditis. The combination of bevacizumab, relatlimab, and ipilimumab may have the potential to further increase the risk of cardiac toxicity.

## 4 Discussion

This study offers three valuable and novel insights into ICI-related cardiac toxicity. First, pairwise meta-analysis evaluated 12 cardiac toxicities, including acute myocardial infarction, acute coronary syndrome, angina pectoris, heart failure, cardiac arrest, sudden cardiac death, atrial fibrillation, sinus tachycardia, ventricular tachycardia, conduction block, bradyarrhythmia, myocarditis, and pericardial effusion. Findings revealed significant associations between ICIs and acute myocardial infarction, cardiac arrest, ventricular tachycardia, and myocarditis. Second, three iterative network meta-analyses of these four cardiac toxicities identified that PD-1 and PD-L1 agents individually or combined confer elevated risk. Specifically, PD-1 (cemiplimab, camrelizumab, nivolumab, pembrolizumab) and PD-L1 (atezolizumab, avelumab, durvalumab) monotherapies were prone to these cardiac toxicities. The third network meta-analysis demonstrated that combination therapies involving nivolumab, pembrolizumab, and avelumab with CTLA-4 and SMTI may further increase cardiac toxicity risk. Hence, cautious use is advised in patients with existing cardiac diseases. Third, disproportionality analysis of the FAERS database confirmed myocarditis risk with monotherapy of PD-1 and PD-L1, and the combination of bevacizumab, relatlimab, and ipilimumab has the potential to further increase the risk of cardiac toxicity. Taken together, PD-1 (nivolumab, pembrolizumab) and PD-L1 (atezolizumab, avelumab) agents, alone or combined, pose risks for cardiac toxicity and warrant careful monitoring in vulnerable patients.

Our study adheres to PRISMA guidelines and is registered in PROSPERO, with findings corroborated by existing literature. We identified four primary cardiac toxicities related to ICIs: acute myocardial infarction, myocarditis, cardiac arrest, and ventricular tachycardia; myocarditis and acute myocardial infarction were especially prevalent, and their incidence increases with combination therapies. Juhasz’s research demonstrated ICIs may heighten thrombosis risk, accelerating vascular atherosclerosis, a precursor to acute myocardial infarction (30). Concurrent lipid-lowering therapy during ICI treatment may confer additional benefit (31). Myocarditis, a rare but potentially fatal complication, has become increasingly recognized with ICI use, particularly with combination regimens—a known risk factor for ICI-mediated myocarditis (32, 33). Mechanistically, PD-1/PD-L1 inhibit phosphatase recruitment, disrupting T-cell activation pathways. The intracellular segment of PD-1 contains ITIM/ITSM motifs, which mainly recruit tyrosine phosphatases SHP-1/2 after ligand binding, thereby inhibiting the activation of the T-cell receptor proximal signaling pathway. Tumor-specific effector T-cell activation causes tumor cell death but also collateral damage to normal tissues. Subsequent antigen presentation of tumor and self-epitopes may trigger autoimmune toxicities (34–37). We found that PD-1/PD-L1 may promote cardiac injury progression via downstream pathways including NLRP3, NF-κB, and STAT1 (38, 39). Prior studies by Sharma (11) found increased myocarditis, pericardial disease, and myocardial infarction risks with ICIs; Zhou (15) identified that ICI monotherapy or combination therapy increases cardiac toxicity, including myocarditis and arrhythmia; Nielsen (13) documented risks of immune-mediated myocarditis with high mortality. However, none applied comprehensive network meta-analysis coupled with FAERS pharmacovigilance validation as in our study.

Although we highlight myocarditis and myocardial infarction as principal cardiac toxicity from ICIs, future research should emphasize two areas: first, multicenter prospective RCTs stratifying patients by baseline cardiac status, smoking, and alcohol use to determine ICIs’ impacts on cardiac toxicity. Four types of events were evaluated: acute myocardial infarction, cardiac arrest, myopathy, and ventricular tachycardia, but it is unclear whether the diagnostic criteria for cardiac toxicity included in the original literature are consistent, so subsequent research should unify the diagnostic criteria for cardiac toxicity. The second one pertains to basic experimental models using PD-1/PD-L1 inhibitors (e.g., pembrolizumab or nivolumab) in rodents, alongside myocardial injury controls (e.g., doxorubicin), employing electrocardiography, CK-MB assays, histology, and immunohistochemistry to elucidate underlying mechanisms of PD-1/PD-L1-induced myocarditis and myocardial infarction, informing clinical management. Our study intends to use a rat/mouse model to confirm our findings; however, pembrolizumab and nivolumab are humanized IgG4 monoclonal antibodies that lack species cross-reactivity with PD-1 molecules in rodents and cannot be directly used for mouse or rat model *in vivo* studies (40). Further studies should use the rat-/mouse-derived anti-PD-1 antibody RMP1.14 or genetically modified models (such as Pdcd1 knockout models) to standardize the *in vivo* study of ICI-induced myocarditis (41, 42). Moreover, future basic research should report cardiac toxicity as a grading of adverse reactions to further determine the degree of impact of ICI on cardiac toxicity.

This study has several limitations. First, it focuses solely on cardiac toxicity associated with ICIs and does not consider the relationship between safety and efficacy; whether better responders have higher adverse event risk remains unaddressed. Future prospective or real-world studies could explore this. Second, although encompassing a large sample, we prioritized incidence proportions and network meta-analysis rankings without adjusting for patient factors (e.g., tumor stage, smoking, alcohol use) potentially influencing cardiac toxicity rates. Third, our study is data-driven, in which the network meta-analysis only applied four types of cardiac toxicity with significant differences in pairwise meta-analysis for subsequent research, and the three-stage network meta-analysis was also data-driven, which may lead to the discovery and amplification of false positives. Fourth, after the three-stage network meta-analysis, although we identified the ICI types and combination therapy types that are most likely to cause cardiac toxicity, we did not consider the issue of drug dosage and did not have sufficient data to conduct a fourth-time network meta-analysis or dose–response-related meta-analysis. Subsequent studies aim to determine the specific PD-1/PD-L1 dosage that is most likely to cause cardiac toxicity. Fifth, the diagnostic criteria for cardiac toxicity such as myocarditis and myocardial infarction in the original RCTs and individual studies from the FAERS database may not be consistent, making it difficult to identify the original source of patient diagnosis, which may result in baseline heterogeneity in our study. Sixth, due to sample size limitations, we did not grade the cardiac toxicity of patients in the network meta-analysis, and the FAERS database lacked basic data for grading and stratifying cardiac toxicity. Therefore, our study did not conduct a stratified analysis of the severity of cardiac toxicity. Seventh, the disproportionality analysis based on the FAERS database revealed that PD-1/PD-L1 monotherapy can increase the risk of myocarditis, especially immune-mediated myocarditis, and the combination therapy of bevacizumab, relatlimab, and ipilimumab may increase the risk of different types of cardiac toxicity. However, there is some inconsistency between the outcomes of disproportionality analysis and network meta-analysis, which may be related to the FAERS database being concentrated in the US population.

In conclusion, ICIs administered alone or in combination significantly increase the risks of acute myocardial infarction, myocarditis, cardiac arrest, and ventricular tachycardia, particularly PD-1 (nivolumab, pembrolizumab) and PD-L1 (atezolizumab, avelumab) agents with elevated myocarditis and acute myocardial infarction risk. These findings underscore the complex cardiac risk profiles of ICI therapies. Despite reshaping cancer treatment paradigms, ICIs necessitate vigilant cardiac toxicity monitoring and comprehensive risk management, especially in PD-1/PD-L1 combination regimens and patients with pre-existing cardiovascular disease.

## Data Availability

The raw data supporting the conclusions of this article will be made available by the authors, without undue reservation.

## Supplementary material

Supplementary Figure 1 Summary risk of bias

Supplementary Figure 2 Network plot of comprehensive intervention type network meta-analysis of cardiac toxicity during the combination and separatation application of immune checkpoint inhibitors with other overall interventions from acute myocardial infarction. The thickness of the lines represents the number of studies, the size of the circles represents the number of participants in this intervention.

Supplementary Figure 3 Local inconsistency of comprehensive intervention type network meta-analysis of cardiac toxicity during the combination and separation application of immune checkpoint inhibitors with other overall interventions from acute myocardial infarction.

Supplementary Figure 4 Network funnel of comprehensive intervention type network meta-analysis of cardiac toxicity during the combination and separation application of immune checkpoint inhibitors with other overall interventions from acute myocardial infarction.

Supplementary Figure 5 Network plot of comprehensive intervention type network meta-analysis of cardiac toxicity during the separation application of specific types of PD-1 and PD-L1 agents from acute myocardial infarction. The thickness of the lines represents the number of studies, the size of the circles represents the number of participants in this intervention.

Supplementary Figure 6 Local inconsistency of comprehensive intervention type network meta-analysis of cardiac toxicity during the separation application of specific types of PD-1 and PD-L1 agents from acute myocardial infarction

Supplementary Figure 7 Network funnel of comprehensive intervention type network meta-analysis of cardiac toxicity during the separation application of specific types of PD-1 and PD-L1 agents from acute myocardial infarction

Supplementary Figure 8 Network plot in comprehensive intervention type network meta-analysis of cardiac toxicity during the combination application of specific types of PD-1 and PD-L1 agents from acute myocardial infarction. The thickness of the lines represents the number of studies, the size of the circles represents the number of participants in this intervention.

Supplementary Figure 9 Local inconsistency of comprehensive intervention type network meta-analysis of cardiac toxicity during the combination application of specific types of PD-1 and PD-L1 agents from acute myocardial infarction

Supplementary Figure 10 Network funnel in comprehensive intervention type network meta-analysis of cardiac toxicity during the combination application of specific types of PD-1 and PD-L1 agents from acute myocardial infarction

Checklist S1 PRISMA Checklist

Diagram S1 PRISMA flow diagram
