# Supplementary material for: Avoidance of cardiac toxicity during the application of immune checkpoint inhibitors: a safety systematic review combining network meta-analysis and pharmacovigilance study
Source: Front Immunol. 2026 Aug 31;17:1919841. doi: 10.3389/fimmu.2026.1919841 (PMC13572159; doi:10.3389/fimmu.2026.1919841)
Supplement: Supplementary file 1 [file Table4.docx]

**Table S3 The clinical characteristics of the patients included in the adverse event (AE) reports for immune checkpoint inhibitors which focus on.**

| **Characteristic** | **Cemiplimab** | **Nivolumab** | **Durvalumab** | **Avelumab** | **Pembrolizumab** | **Atezolizumab** | ***P* value** |
| --- | --- | --- | --- | --- | --- | --- | --- |
| **Overall reports(n)** | 2528 | 68184 | 15868 | 2327 | 60057 | 25301 |  |
| **Sex – no. (%)** |  |  |  |  |  |  |  |
| Female | 628 (24.8%) | 20592 (30.2%) | 4591 (28.9%) | 607 (26.1%) | 27539 (45.9%) | 7969 (31.5%) | 0.199 |
| Male | 887 (35.1%) | 38226 (56.1%) | 8924 (56.2%) | 1509 (64.8%) | 29223 (48.7%) | 13879 (54.9%) |  |
| Missing | 1013 (40.1%) | 9366 (13.7%) | 2353 (14.8%) | 211 (9.1%) | 3295 (5.5%) | 3453 (13.6%) |  |
| **Age(year,%)** |  |  |  |  |  |  |  |
| <2 | - | 22 (0.0%) | 6 (0.0%) | - | 18 (0.0%) | 6 (0.0%) | 0.088 |
| 2~11 | 1 (0.0%) | 101 (0.1%) | 2 (0.0%) | - | 29 (0.0%) | - |  |
| 12~17 | 1 (0.0%) | 144 (0.2%) | - | 2 (0.1%) | 56 (0.1%) | 14 (0.1%) |  |
| 18~64 | 261 (10.3%) | 21606 (31.7%) | 3533 (22.3%) | 550 (23.6%) | 17437 (29.0%) | 7891 (31.2%) |  |
| 65~85 | 501 (19.8%) | 25248 (37.0%) | 5844 (36.8%) | 1177 (50.6%) | 24322 (40.5%) | 11213 (44.3%) |  |
| >85 | 98 (3.9%) | 968 (1.4%) | 157 (1.0%) | 84 (3.6%) | 1316 (2.2%) | 555 (2.2%) |  |
| Missing | 1666 (65.9%) | 20095 (29.5%) |  |  | 16879 (28.1%) | 5615 (22.2%) |  |
| **Weight(kg,%)** |  |  |  |  |  |  |  |
| ＜50 | 5 (0.2%) | 1859 (2.7%) | 415 (2.6%) | 67 (2.9%) | 1694 (2.8%) | 867 (3.4%) | 0.090 |
| 50～100 | 8 (0.3%) | 1648 (2.4%) | 156 (1.0%) | 83 (3.6%) | 731 (1.2%) | 499 (2.0%) |  |
| ＞100 | 48 (1.9%) | 15325 (22.5%) | 3535 (22.3%) | 705 (30.3%) | 10163 (16.9%) | 7450 (29.4%) |  |
| Missing | 2467 (97.6%) | 49352 (72.4%) | 11762 (74.1%) | 1472 (63.3%) | 47469 (79.0%) | 16485 (65.2%) |  |
| **Reporter type** |  |  |  |  |  |  |  |
| Consumer(CN) | 462 (18.3%) | 16274 (23.9%) | 2014 (12.7%) | 203 (8.7%) | 18259 (30.4%) | 3375 (13.3%) | 0.224 |
| Health Professional(HP) | 334 (13.2%) | 12505 (18.3%) | 972 (6.1%) | 162 (7.0%) | 8329 (13.9%) | 2138 (8.5%) |  |
| Pharmacist(PH) | 188 (7.4%) | 4530 (6.6%) | 585 (3.7%) | 152 (6.5%) | 4016 (6.7%) | 1169 (4.6%) |  |
| Physician(MD) | 1506 (59.6%) | 21714 (31.8%) | 9100 (57.3%) | 1628 (70.0%) | 26713 (44.5%) | 17789 (70.3%) |  |
| Missing | 38 (1.5%) | 13161 (19.3%) | 3197 (20.1%) | 182 (7.8%) | 2740 (4.6%) | 830 (3.3%) |  |
| **Serious or not(%)** |  |  |  |  |  |  |  |
| NO | 356 (14.1%) | 7726 (11.3%) | 808 (5.1%) | 290 (12.5%) | 10054 (16.7%) | 1239 (4.9%) | 0.199 |
| YES | 2172 (85.9%) | 60458 (88.7%) | 15060 (94.9%) | 2037 (87.5%) | 50003 (83.3%) | 24062 (95.1%) |  |
| **Fatal or not(%)** |  |  |  |  |  |  |  |
| NO | 2202 (87.1%) | 57894 (84.9%) | 11319 (71.3%) | 1808 (77.7%) | 52163 (86.9%) | 19654 (77.7%) | 0.199 |
| YES | 326 (12.9%) | 10290 (15.1%) | 4549 (28.7%) | 519 (22.3%) | 7894 (13.1%) | 5647 (22.3%) |  |
| **Outcome type** |  |  |  |  |  |  |  |
| Death(DE) | 326 (12.9%) | 10290 (15.1%) | 4549 (28.7%) | 519 (22.3%) | 7894 (13.1%) | 5647 (22.3%) | 0.220 |
| Life-threatening(LT) | 58 (2.3%) | 1484 (2.2%) | 555 (3.5%) | 63 (2.7%) | 1211 (2.0%) | 499 (2.0%) |  |
| Disability(DS) | 27 (1.1%) | 537 (0.8%) | 142 (0.9%) | 14 (0.6%) | 879 (1.5%) | 199 (0.8%) |  |
| Hospitalization(HO) | 731 (28.9%) | 15626 (22.9%) | 3425 (21.6%) | 700 (30.1%) | 14232 (23.7%) | 8869 (35.1%) |  |
