# Supplementary material for: Avoidance of cardiac toxicity during the application of immune checkpoint inhibitors: a safety systematic review combining network meta-analysis and pharmacovigilance study
Source: Front Immunol. 2026 Aug 31;17:1919841. doi: 10.3389/fimmu.2026.1919841 (PMC13572159; doi:10.3389/fimmu.2026.1919841)
Supplement: Supplementary file 2 [file Table1.doc]

**Table S1 Search strategy**

(((((((((Immune checkpoint inhibitors) OR (PD-1)) OR (PD-L1)) OR (CTLA-4) AND (Randomized controlled trial)) OR (Clinical trial)) AND (Adverse reaction)) OR (Side effect)) OR (Cardiactoxicity)) )

**Keywords**

Immune checkpoint inhibitors

PD-1

PD-L1

CTLA-4

Randomized controlled trial

Clinical trial

Adverse reaction

Side effect

Cardiactoxicity
