# Supplementary material for: Avoidance of cardiac toxicity during the application of immune checkpoint inhibitors: a safety systematic review combining network meta-analysis and pharmacovigilance study
Source: Front Immunol. 2026 Aug 31;17:1919841. doi: 10.3389/fimmu.2026.1919841 (PMC13572159; doi:10.3389/fimmu.2026.1919841)
Supplement: Supplementary file 3 [file Table2.doc]

**Table S2 Drug-name standardization in FAERS databases**

"CEMIPLIMAB", "LIBTAYO", "LIBTAYO [CEMIPLIMAB]",

"NIVOLUMAB", "BLINDED: NIVOLUMAB", "BLINDED NIVOLUMAB", "NIVOLUMAB BMS", "OPDIVO",

"DURVALUMAB", "DURVALUMAB.", "MEDI4736(DURVALUMAB)", "IMFINZI",

"AVELUMAB", "BAVENCIO",

"PEMBROLIZUMAB", "PEMBROLIZUMAB 100MG/4ML MERCK", "PEMBROLIZUMAB 200MG MERCK RESEARCH LAB", "PEMBROLIZUMAB, 100MG/4ML MERCK", "PEMBROLIZUMAB (MK-3475)", "BLINDED PEMBROLIZUMAB", "PEMBROLIZUMAB 200 MG", "MK-3475 (PEMBROLIZUMAB) 200MG IV", "PEMBROLIZUMAB 200 MG", "PEMBROLIZUMAB", "MK-3475 PEMBROLIZUMAB 100MG", "KEYTRUDA (PEMBROLIZUMAB)", "MK-3475", "MK-3475 SOLUTION FOR INFUSION", "BLINDED MK-3475", "KEYTRUDA",

"ATEZOLIZUMAB", "ATEZOLIZUMAB.", "MPDL3280A (ATEZOLIZUMAB)", "BLINDED ATEZOLIZUMAB", "ATEZOLIZUMAB 1200MG", "ATEZOLIZUMAB (GENETICAL RECOMBINATION)", "TECENTRIQ", "TECENTRIQ (ATU)",

"CAMRELIZUMAB", "CAMRELIZUMAB FOR INJECTION"
