# Supplementary material for: Avoidance of cardiac toxicity during the application of immune checkpoint inhibitors: a safety systematic review combining network meta-analysis and pharmacovigilance study
Source: Front Immunol. 2026 Aug 31;17:1919841. doi: 10.3389/fimmu.2026.1919841 (PMC13572159; doi:10.3389/fimmu.2026.1919841)
Supplement: Supplementary file 4 [file Table3.doc]

**Table S3 Baseline characteristic of included studies**

|  |  |  |  | **Intervention** |  |  |  | **Control** |  |  |  |
| --- | --- | --- | --- | --- | --- | --- | --- | --- | --- | --- | --- |
| **First author, year** | **Cancer type** | **Cancer stage** | **No.Clinical Trial** | **Sample size(gender M/F)** | **ICI type** | **ICI agent** | **ICI dose** | **Sample size(gender M/F)** | **Treatment type** | **Treatment agent** | **Treatment dose** |
| André T, 2025[1] | CRC | Late stage | NCT04008030 | 354(162/192) | PD-1+CTLA-4 | Nivolumab +ipilimumab | 240 mg+ 1 mg/kg | 353(190/163) | PD-1 | Nivolumab | 240 mg |
| Antonia SJ, 2017[2] | NSCLC | III stage | NCT02125461 | 476(334/142) | PD-L1 | Durvalumab | 10mg/kg | 237(166/71) | Placebo |  |  |
| Antoniotti C, 2022[3] | CRC | Late stage | NCT03721653 | 145(83/62) | PD-L1+chemotherapy | Atezolizumab+Bevacizumab+FOLFOXIRI | 5 mg/kg | 73(42/31) | SMTI+chemotherapy | Bevacizumab+FOLFOXIRI | 840mg |
| Barlesi F, 2018[4] | NSCLC | IIIB-IV | NCT02395172 | 396(269/127) | PD-L1 | Avelumab | 10 mg/kg | 396(273/123) | Chemotherapy | Docetaxel |  |
| Bellmunt J, 2021[5] | Bladder cancer | pT2 pT3/4 | NCT02450331 | 406(322/84) | PD-L1 | Atezolizumab | 1200 mg | 403(316/87) | Usual care |  |  |
| Borghaei H, 2023[6] | NSCLC | IV | NCT02477826 | 377(264/113) | PD-1+chemotherapy | Nivolumab | 360 mg | 378(266/112) | Chemotherapy | platinum-based |  |
| Cho BC, 2023[7] | NSCLC | Late stage | NCT03631706 | 152(116/36) | PD-1 | Pembrolizumab | 200 mg | 152(110/42) | Bifunctional fusion protein | Bintrafusp Alfa |  |
| Choudhury AD, 2024[8] | Prostate cancer | Late stage | NCT03093428 | 69(69/0) | PD-1+radiotherapy | Pembrolizumab+Radium-223 | 200 mg | 13(13/0) | Radiotherapy | Radium-223 |  |
| Choueiri TK, 2024[9] | RCC | Late stage | NCT02684006 | 442(316/16) | PD-1+SMTI | Avelumab + axitinib | 10 mg/kg | 444(344/100) | SMTI | Sunitinib |  |
| Colombo N, 2024[10] | Endometrial cancer | III-IV | NCT03603184 | 360(0/360) | PD-L1+chemotherapy | Atezolizumab+carboplatin+paclitaxel | 1200 mg | 189(0/189) | Chemotherapy | Carboplatin+paclitaxel |  |
| Diaz LA, 2022[11] | CRC | Late stage | NCT02563002 | 153(71/82) | PD-1 | Pembrolizumab | 200mg | 154(82/72) | Chemotherapy | mFOLFOX6/FOLFIRI/Bevacizumab |  |
| Eggermont AM, 2015[12] | Melanoma | III | NCT00636168 | 475(296/179) | CTLA-4 | Ipilimumab | 10 mg/kg | 476(293/183) | Placebo |  |  |
| Eggermont AM, 2018[13] | Melanoma | III | NCT02362594 | 514(324/190) | PD-1 | Pembrolizumab | 200 mg | 505(304/201) | Placebo |  |  |
| Eskander RN, 2025[14] | Endometrial cancer | III-IV | NCT03914612 | 405(0/405) | PD-1+chemotherapy | Pembrolizumab+ paclitaxel–carboplatin | 200 mg | 408(0/408) | Placebo+chemotherapy | Placebo+paclitaxel–carboplatin |  |
| Fehrenbacher L, 2016[15] | NSCLC | Late stage | NCT01903993 | 144(92/51) | PD-L1 | Atezolizumab | 1200mg | 143(76/67) | Chemotherapy | Docetaxel |  |
| Felip E, 2021[16] | NSCLC | IB-IIIA | NCT02486718 | 507(337/170) | PD-L1+chemotherapy | Atezolizumab+Platinum-based | 1200mg | 498(335/163) | Chemotherapy | Platinum-based |  |
| Felip E, 2025[17] | Mesothelioma of pleura | Ⅰ-Ⅳ | NCT03762018 | 200(160/40) | PD-L1+SMTI+chemotherapy | Atezolizumab+Bevacizumab+Carboplatin+Pemetrexed | 1200mg | 200(156/44) | SMTI+chemotherapy | Bevacizumab+Carboplatin+Pemetrexed |  |
| Garassino M, 2023[18] | NSCLC | Ⅳ | NCT02578680 | 410(254/156) | PD-1+chemotherapy | Pembrolizumab +Pemetrexed +Platinum | 200 mg | 206(109/97) | Placebo+chemotherapy | Pemetrexed +Platinum |  |
| Gettinger SN, 2021[19] | NSCLC | Ⅳ | NCT02785952 | 125(83/42) | PD-1+CTLA-4 | Nivolumab +Ipilimumab | 3 mg/kg+ 1 mg/kg | 127(86/41) | PD-1 | Nivolumab | 3 mg/kg |
| Goldman JW, 2021[20] | SCLC | Ⅰ-Ⅳ | NCT03043872 | 268(202/66) | PD-L1+CTLA-4+chemotherapy | Durvalumab+Tremelimumab+Platinum-Etoposide | 1500 mg+ 75 mg | 268(190/78) | PD-L1+ Platinum-Etoposide | Durvalumab+Platinum-Etoposide | 1500 mg |
|  |  |  |  |  |  |  |  | 269(184/85) | Platinum-Etoposide | Platinum-Etoposide |  |
| Herbst RS, 2015[21] | NSCLC | ⅢB-Ⅳ | NCT01905657 | 345(212/133) | PD-1 | Pembrolizumab | 2 mg/kg | 343(209/134) | Chemotherapy | Docetaxel |  |
|  |  |  |  | 346(213/133) | PD-1 | Pembrolizumab | 10 mg/kg |  |  |  |  |
| Hodi FS, 2016[22] | Melanoma | III-IV | NCT01927419 | 95(63/32) | PD-1+CTLA-4 | Nivolumab +ipilimumab | 1 mg/kg 3 mg/kg | 47(32/15) | CTLA-4+placebo | Ipilimumab+placebo | 3 mg/kg |
| Hodi FS, 2018[23] | Melanoma | III-IV | NCT01844505 | 314(206/108) | PD-1+CTLA-4 | Nivolumab +ipilimumab | 1 mg/kg 3 mg/kg | 316(202/114) | PD-1 | Nivolumab | 1 mg/kg |
|  |  |  |  |  |  |  |  | 315(202/113) | CTLA-4 | Ipilimumab | 3 mg/kg |
| Huang J, 2020[24] | Esophageal squamous cell carcinoma | Late stage | NCT03099382 | 228(208/20) | PD-1 | Camrelizumab | 200 mg | 220(192/28) | Chemotherapy | Docetaxel/ irinotecan |  |
| Janjigian YY, 2021[25] | ER2-positive gastric cancer | IV | NCT03615326 | 217(179/38) | PD-1+SMTI+chemotherapy | Pembrolizumab+trastuzumab +5-fluorouracil+cisplatin /capecitabine+oxaliplatin | 200 mg, loading dose 8 mg/kg, maintenance dose 6 mg/kg | 217(174/43) | Placebo+SMTI+chemotherapy | Placebo+trastuzumab +5-fluorouracil+cisplatin /capecitabine+oxaliplatin |  |
| Johnson ML, 2022[26] | NSCLC | IV | NCT03164616 | 338(269/69) | CTLA-4+PD-L1+chemotherapy | Durvalumab+Tremelimumab+ platinum-based | 75mg 1500 mg | 338(253/85) | PD-L1+ chemotherapy | Durvalumab+ platinum-based | 75mg |
|  |  |  |  |  |  |  |  | 337(248/89) | Chemotherapy | platinum-based |  |
| Kim Y, 2025[27] | Cervical cancer | IV | NCT03635567 | 57(0/57) | PD-1+chemotherapy | Pembrolizumab+cisplatin /carboplatin | 200 mg | 40(0/40) | Placebo+chemotherapy | Paclitaxel  +cisplatin /carboplatin |  |
| Kojima T, 2020[28] | Esophagus cancer | Late stage | NCT02564263 | 314(273/41) | PD-1 | Pembrolizumab | 200 mg | 314(271/42) | Chemotherapy | paclitaxel  and cisplatin or carboplatin |  |
| Langer CJ, 2016[29] | NSCLC | ⅢB-Ⅳ | NCT02039674 | 60(22/38) | PD-1 | Pembrolizumab | 200 mg | 63(26/37) | Chemotherapy | carboplatin +pemetrexed |  |
| Lenz H, 2024[30] | CRC | Late stage | NCT03414983 | 127(70/57) | PD-1+SMTI+chemotherapy | Nivolumab+Bevacizumab+mFOLFOX6 | 240 mg | 68(49/19) | SMTI+chemotherapy | Bevacizumab+mFOLFOX6 |  |
| Lu S, 2024[31] | NSCLC | Late stage | NCT03663205 | 223(168/55) | PD-1+chemotherapy | Tislelizumab+platinum-based | 200mg | 111(79/32) | Chemotherapy | platinum-based |  |
| Luke JJ, 2024[32] | Melanoma | ⅡB-ⅡC | NCT03553836 | 487(300/187) | PD-1 | Pembrolizumab | 200 mg |  | Placebo |  |  |
| Machiels J, 2024[33] | Head and neck squamous cell carcinoma | Ⅲ-Ⅳ | NCT03040999 | 402(331/71) | PD-1+chemotherapy | Pembrolizumab+ cisplatin | 200 mg | 402(329/73) | Placebo+chemotherapy | cisplatin |  |
| Mai H, 2022[34] | Nasopharyngeal carcinoma | Ⅰ-Ⅳ | NCT03581786 | 146(124/22) | PD-1+chemotherapy | Toripalimab+chemotherapy | 240 mg | 143(116/27) | Placebo+chemotherapy |  |  |
| Maio M, 2017[35] | malignant mesothelioma | Ⅲ-Ⅳ | NCT01843374 | 382(283/99) | CTLA-4 | Tremelimumab | 10mg/kg | 189(151/38) | Placebo |  |  |
| Mell LK, 2024[36] | Head and neck squamous cell carcinoma | I-Ⅳ | NCT03258554 | 123(102/21) | PD-L1+radiotherapy | Durvalumab+radiotherapy | 1500 mg | 63(54/9) | radiotherapy+SMTI | Cetuximab |  |
| Mok TS, 2019[37] | NSCLC | III-IV | NCT02220894 | 637(450/187) | PD-1 | Pembrolizumab | 200 mg | 637(452/185) | Chemotherapy | platinum-based |  |
| Monk BJ, 2021[38] | Epithelial ovarian cancer, fallopian tube cancer, or peritoneal cancer | Ⅲ-Ⅳ | NCT02718417 | 332(0/332) | PD-L1+ chemotherapy | Avelumab+carboplatin | 10 mg/kg | 335(0/335) | Chemotherapy | Carboplatin |  |
| Moore KN, 2021[39] | Epithelial ovarian cancer, fallopian tube cancer or peritoneal cancer | Ⅲ-Ⅳ | NCT03038100 | 651(0/651) | PD-L1+SMTIR+Chemotherapy | Atezolizumab+Bevacizumab+Paclitaxel+Carboplatin | 1200 mg+ 15 mg/kg | 650(0/650) | Placebo+SMTI+Chemotherapy | Placebo+Bevacizumab+Paclitaxel+Carboplatin | 15 mg/kg |
| Motzer RJ, 2019[40] | RCC | Late stage | NCT02231749 | 550(413/137) | PD-1+CTLA-4 | Nivolumab +ipilimumab | 3 mg/kg 1 mg/kg | 546(395/151) | SMTI | Sunitinib |  |
| Felip E, 2025[41] | NSCLC | IB- IIIA | NCT02486718 | 507(337/170) | PD-L1 | Atezolizumab | 1200 mg | 498(335/163) | Usual care |  |  |
| Zhou A, 2025[42] | RCC | Late stage | NCT04523272 | 264(204/60) | PD-L1＋SMTI | Benmelstobart+Anlotinib | 1200 mg | 264(196/67) | SMTI | Sunitinib |  |
| Zeidan AM, 2022[43] | AML | New AML | NCT02775903 | 64(40/24) | PD-L1＋chemoterapy | Durvalumab+Azacitidine | 1500mg | 65(31/34) | Chemotherapy | Azacitidine |  |
| Baas P, 2021[44] | Malignant pleural mesothelioma | I-Ⅳ | NCT02899299 | 303(234/69) | PD-1+CTLA-4 | Nivolumab +Ipilimumab | 3 mg/kg＋ 1 mg/kg | 302(233/69) | Chemotherapy | Pemetrexed+cisplatin |  |
| Yang JC, 2024[45] | NSCLC | IV | NCT03515837 | 245(93/152) | PD-1+chemotherapy | Pembrolizumab  +Pemetrexed+Platinum | 200mg | 247(96/151) | Placebo+chemotherapy | Pemetrexed+Platinum |  |
| Xu J, 2023[46] | Esophageal squamous cancer | Late stage | NCT03783442 | 326(282/44) | PD-1+chemotherapy | Tislelizumab+Cisplat/oxaliplatin +fluoropyrimidine | 200mg | 323(281/42) | Placebo+chemotherapy | Cisplat/oxaliplatin +fluoropyrimidine |  |
| Xing W, 2021[47] | esophageal squamous cancer | II/III/IVa | NCT03985670 | 15(13/2) | PD-1+chemotherapy | Toripalimab+Paclitaxel+Cisplatin | 240mg | 15(9/6) | Placebo+chemotherapy | Paclitaxel+Cisplatin |  |
| Xiao W, 2024[48] | CRC | Late stage | [NCT04304209](http://clinicaltrials.gov/show/NCT04304209) | 67(43/24) | PD-1+chemotherapy | Sintilimab+Oxaliplatin/capecitabine | 200mg | 67(37/30) | Placebo+chemotherapy | Oxaliplatin/capecitabine |  |
| Winer EP, 2021[49] | BC | I-IV | NCT02555657 | 312(0/312) | PD-1 | Pembrolizumab | 200 mg | 314(2/312) | Chemotherapy | Capecitabine/eribulin/Gemcitabine/vinorelbine |  |
| West H, 2019[50] | NSCLC | IV | NCT02367781 | 433(227/206) | PD-L1+ chemotherapy | Atezolizumab+Carboplatin +nab-paclitaxel | 1200mg | 240(138/102) | Chemotherapy | Carboplatin +nab-paclitaxel |  |
| Vano Y, 2022[51] | RCC | IV | NCT02960906 | 101(83/18) | PD-1+CTLA-4 | Nivolumab +ipilimumab | 3 mg/kg 1 mg/kg | 40 | SMTI | sunitinib /pazopanib |  |
|  |  |  |  | 58 | PD-1 | Nivolumab | 3 mg/kg |  |  |  |  |
| Usmani SZ, 2019[52] | Multiple myeloma | I | NCT02579863 | 151(70/81) | PD-1+ chemotherapy | Pembrolizumab+lenalidomide+dexamethasone | 200mg | 150(71/79) | Chemotherapy | Lenalidomide+dexamethasone |  |
| Tian F, 2025[53] | CRC | T3-T4 | ChiCTR2100052288 | 49(33/16) | PD-1+chemotherapy | Sintilimab+Capecitabine+oxaliplatin | 200 mg/m2 | 49(35/14) | Chemotherapy | Capecitabine/oxaliplatin |  |
| Takahashi M, 2023[54] | Breast cancer | Late stage | NCT03036488 | 136(0/136) | PD-1＋chemotherapy | Pembrolizumab+carboplatin +paclitaxel | 200mg | 80(0/80) | Chemotherapy | carboplatin +paclitaxel |  |
| Sun L, 2025[55] | Gastric cancer | Late stage | NCT04294784 | 30(15/15) | PD-1+chemotherapy | Camrelizumab+Nab-paclitaxel | 200mg | 28(16/12) | Chemotherapy | Nab-paclitaxel |  |
| Spicer JD, 2024[56] | NSCLC | II-IIIB | NCT03425643 | 397(279/118) | PD-1+chemotheraoy | Pembrolizumab+cisplatin-based | 200mg | 400 | Placebo+chemotherapy | Cisplatin-based |  |
| Sezer A, 2021[57] | NSCLC | IIIB-IV | NCT03088540 | 664(560/104) | PD-1 | Cemiplimab | 350mg | 634(525/109) | Chemotherapy | Cemiplimab/platinum |  |
| Scherpereel A, 2019[58] | Malignant pleural mesothelioma | III-IV | NCT02716272 | 63(47/16) | PD-1+CTLA-4 | Nivolumab +  ipilimumab | 3 mg/kg+1 mg/kg | 62(53/9) | PD-1 | Nivolumab | 3 mg/kg |
| Rubinstein MM, 2022[59] | Endometrial cancer | I-IV | NCT03015129 | 39(0/39) | PD-L1+ CTLA-4 | Durvalumab +tremelimumab | 1500 mg+75 mg every 4 weeks | 38(0/38) | PD-L1 | Durvalumab | 1500 mg |
| Rini BI, 2022[60] | RCC | Late stage | NCT02684006 | 442(316/126) | PD-L1＋SMTI | Avelumab +axitinib | 10mg/kg | 444(344/100) | SMTI | Sunitinib |  |
| Ribas A, 2013[61] | Melanoma | III-IV | NCT00257205 | 428(248/180) | CTLA-4 | Tremelimumab | 15 mg/kg | 327(238/189) | Usual care |  |  |
| Rha SY, 2023[62] | Adenocarcinoma of the stomach or gastroesophageal junction | I-IV | NCT03675737 | 790(527/263) | PD-1+chemotheraoy | Pembrolizumab+Fluorouracil /cisplatin /capecitabine /oxaliplatin | 200mg | 789(544/245) | Placebo+chemotherapy | Fluorouracil /cisplatin /capecitabine /oxaliplatin |  |
| Reckamp KL, 2022[63] | NSCLC | III-IV | NCT03971474 | 69(41/28) | PD-1+SMTI | Pembrolizumab+Ramucirumab | 200 mg | 67(42/25) | Usual care |  |  |
| Özgüroğlu M, 2023[64] | NSCLC | Late stage | NCT03088540 | 357(313/44) | PD-1 | Cemiplimab | 350 mg | 355(294/61) | Chemotherapy | Platinum-based |  |
| Paz-Ares L, 2022[65] | NSCLC | IIB-IIII | NCT02504372 | 590(401/189) | PD-1 | Pembrolizumab | 200 mg | 587(403/184) | Placebo |  |  |
| Nishio M, 2020[66] | NSCLC | IV | NCT02657434 | 292(192/100) | PD-L1＋chemoterapy | Atezolizumab+Carboplatin /cisplatin +  pemetrexed | 1200 mg | 286(192/94) | Chemotherapy | Carboplatin /cisplatin +  pemetrexed |  |
| Powles T, 2022[67] | RCC | Late stage | NCT03142334 | 496(347/149) | PD-1 | Pembrolizumab | 200 mg | 498(359/139) | Placebo |  |  |
| Powles T, 2020[68] | RCC | Late stage | NCT02853331 | 432(308/124) | PD-1＋SMTI | Pembrolizumab + axitinib | 200 mg | 429(320/109) | SMTI | Sunitinib |  |
| Pignata S, 2023[69] | Endometrial cancer | Late stage | NCT03503786 | 63(0/63) | PD-L1+ chemotherapy | Avelumab+Carboplatin + paclitaxel | 10 mg/kg | 62(0/62) | Chemotherapy | Carboplatin + paclitaxel |  |
| Paz-Ares L, 2025[70] | SCLC | I-IV | NCT05091567 | 242(151/91) | PD-L1+ chemotherapy | Atezolizumab+Lurbinectedin | 1200mg | 241(151/90) | Chemotherapy | Lurbinectedin |  |
| Long GV, 2024[71] | Melanoma | III-IV | NCT01866319 | 278(162/116) | CTLA-4 | Ipilimumab | 3 mg/kg | 279(161/118) | PD-1 | Pembrolizumab | 10mg/kg Q2W |
|  |  |  |  |  |  |  |  | 277(174/103) | PD-1 | Pembrolizumab | 10mg/kg Q3W |
| NCT03147287[72] | Breast cancer | I-IV | NCT03147287 | 54(0/54) | PD-L1+SMTI+chemotherapy | Avelumab+Palbociclib+Fulvestrant | - | 111(2/109) | SMTI+chemotherapy | Palbociclib+Fulvestrant |  |
|  |  |  |  |  |  |  |  | 55(0/55) | Chemotherapy | Fulvestrant |  |
| NCT03906071[73] | SCLC | I-IV | NCT03906071 | 284(170/114) | PD-1+SMTI | Nivolumab+Sitravatinib | 240mg every 2 weeks or 480mg every 4 weeks | 293(168/125) | Chemotherapy | Docetaxel |  |
| NCT03055013[74] | RCC | T2-T4 | NCT03055013 | 381(268/113) | PD-1+Surgery | Nivolumab | 480mg | 399(277/122) | Surgery |  |  |
| Ascierto PA, 2019[75] | Melanoma | I-IV | NCT02130466 | 60(33/27) | PD-1+SMTI | Pembrolizumab +Dabrafenib+trametinib | 2 mg/kg | 60(36/24) | SMTI | Dabrafenib+trametinib |  |
| Bang Y, 2017[76] | Gastric cancer or gastric esophageal junction cancer | Late stage | NCT01585987 | 57(36/21) | CTLA-4 | Ipilimumab | 10mg/kg | 57(41/16) | Usual care |  |  |
| Cella D, 2019[77] | RCC | Late stage | NCT02231749 | 550(413/137) | PD-1+CTLA-4 | Nivolumab +ipilimumab | 3 mg/kg 1 mg/kg | 546(395/151) | SMTI | sunitinib |  |
| Motzer RJ, 2020[78] | RCC | Late stage | NCT01668784 | 410(315/90) | PD-1 | Nivolumab | 3 mg/kg | 411(304/107) | Chemotherapy | Everolimus |  |
| Cohen EE, 2018[79] | Head and neck squamous cell carcinoma | Late stage | NCT02252042 | 247(207/40) | PD-1 | Pembrolizumab | 200mg | 248(205/43) | Chemotherapy | Methotrexate/docetaxel/cetuximab |  |
| Cortes J, 2020[80] | Breast cancer | IV | NCT02819518 | 566(0/566) | PD-1+ chemotherapy | Pembrolizumab+nab-paclitaxel/paclitaxel/gemcitabine+ carboplatin | 200mg | 281(0/281) | Placebo+chemotherapy | Nab-paclitaxel/paclitaxel/gemcitabine+ carboplatin |  |
| Emens LA 2020[81] | Breast cancer | Late stage | NCT02924883 | 133(2/131) | PD-L1+chemotherapy | Atezolizumab+Trastuzumab | 1200mg+3.6mg/kg | 69(0/69) | Chemotherapy | Trastuzumab |  |
| NCT03662659[82] | Adenocarcinoma of the gastroesophageal junction | Late stage | NCT03662659 | 138(94/44) | PD-1+LAG-3+ chemotherapy | Nivolumab+BMS-986213+ XELOX | - | 136(98/38) | PD-1+ chemotherapy | Nivolumab+XELOX |  |
| Kato K, 2019[83] | Esophageal squamous cell carcinoma | IV | NCT02569242 | 210(179/31) | PD-1 | Nivolumab | 240mg | 209(185/24) | Chemotherapy | Paclitaxel /docetaxel |  |
| Llovet JM, 2023[84] | HCC | Late stage | NCT03713593 | 395(317/78) | PD-1+ SMTI | Pembrolizumab+Lenvatinib | 200 mg | 399(327/72) | SMTI | Lenvatinib |  |
| NCT02752074[85] | Melanoma | III-IV | NCT02752074 | 354(217/137) | PD-1+IDOi | Pembrolizumab + epacadostat | - | 352(206/146) | PD-1+Placebo | Pembrolizumab+Placebo |  |
| Marth C, 2024[86] | Endometrial cancer | III-IV | NCT03884101 | 420(0/420) | PD-1+ SMTI | Pembrolizumab+Lenvatinib | 200 mg | 422(0/422) | Chemotherapy | Paclitaxel  +carboplatin |  |
| Monk BJ, 2021[87] | Epithelial ovarian cancer, fallopian tube cancer or peritoneal cancer | III-IV | NCT02718417 | 332(0/332) | PD-L1+ chemotherapy | Avelumab+Carboplatin + paclitaxel | 10 mg/kg | 335(0/335) | Chemotherapy | Carboplatin + paclitaxel |  |
| Motzer RJ, 2022[88] | RCC | IV | NCT03141177 | 323(249/74) | PD-1+ SMTI | Nivolumab +cabozantinib | 240 mg | 328(232/96) | SMTI | Sunitinib |  |
| Eng C, 2019[89] | CRC | IV | NCT02788279 | 183(107/76) | PD-L1+SMTI | Atezolizumab +cobimetinib | 840mg+60 mg | 90(51/39) | SMTI | Regorafenib |  |
|  |  |  |  | 90(59/31) | PD-L1 | Atezolizumab | 840mg |  |  |  |  |
| Hodi FS, 2010[90] | Melanoma | III | NCT00094653 | 403(247/156) | CTLA4+GP100 | Ipilimumab +Glycoprotein 100 | 3 mg/kg | 137(81/56) | CTLA4 | Ipilimumab |  |
|  |  |  |  |  |  |  |  | 136(73/63) | GP100 | Glycoprotein 100 |  |
| Robert C, 2011[91] | Melanoma | III | NCT00324155 | 250(152/98) | CTLA-4+chemotherapy | Ipilimumab | 10 mg/kg+850 mg/m^2 | 252(149/103) | Placebo+chemotherapy | Dacarbazine |  |
| Reck M, 2012[92] | SCLC | I-IV | NCT00527735 | 85(65/20) | CTLA-4+chemotherapy | Ipilimumab | 10 mg/kg | 45(33/12) | Placebo+chemotherapy | Paclitaxel +carboplatin |  |
| Lynch TJ, 2012[93] | NSCLC | IIIB/IV | NCT00527735 | 138(102/36) | CTLA-4+chemotherapy | Ipilimumab | 10 mg/kg | 66(49/17) | Placebo+chemotherapy | Placebo + Paclitaxel/Carboplatin |  |
| Kwon ED, 2014[94] | PC | III | NCT00861614 | 399(399/0) | CTLA-4+radiotherapy | Ipilimumab+radiotherapy | 10 mg/kg | 400(400/0) | Placebo+radiotherapy |  |  |
| Beer TM, 2016[95] | PC | III | NCT01057810 | 400(400/0) | CTLA-4 | Ipilimumab | 10 mg/kg | 202(202/0) | Placebo |  |  |
| Govindan R, 2017[96] | NSCLC | IV | NCT01285609 | 388(326/62) | CTLA-4+chemotherapy | Ipilimumab +Paclitaxel/Carboplatin |  | 361(309/52) | Placebo+chemotherapy | Paclitaxel/Carboplatin |  |
| Kang Y, 2017[97] | Gastric cancer | Late stage | NCT02267343 | 330(229/101) | PD-1 | Nivolumab | 3 mg/kg | 163(119/44) | Placebo |  |  |
| Emens LA, 2021[98] | BC | Late stage | NCT02425891 | 451(2/449) | PD-L1+chemotherapy | Atezolizumab+Nab-Paclitaxel | 840 mg | 451(1/450) | Placebo+chemotherapy | Nab-Paclitaxel |  |
| Shitara K, 2020[99] | Gastric cancer | Late stage | NCT02494583 | 257(195/62) | PD-1+chemotherapy | Pembrolizumab+Cisplatin +fluoroura/capecitabine | 200 mg | 250(179/71) | Chemotherapy | Cisplatin +fluoroura/capecitabine |  |
|  |  |  |  | 256(180/76) | PD-1 | Pembrolizumab | 200 mg |  |  |  |  |
| Galsky MD, 2020[100] | Urothelial Cancer | Late stage | NCT02500121 | 55(39/16) | PD-1 | Pembrolizumab | 200 mg | 52(42/10) | Placebo |  |  |
| Owonikoko TK, 2021[101] | SCLC | I-IV | NCT02538666 | 303(202/101) | CTLA-4+PD-1 | Nivolumab+ Ipilimumab | 1 mg/kg 3 mg/kg | 304(199/105) | Placebo |  |  |
| Bajorin DF, 2021[102] | Urothelial Carcinoma | I-IV | NCT02632409 | 353(265/88) | PD-1 | Nivolumab | 240mg | 356(275/81) | Placebo |  |  |
| Lim M, 2022[103] | Glioblastoma | New diagnose | NCT02667587 | 358(205/153) | PD-1+chemotherapy+radiotherapy | Nivolumab+Temozolomide+radiotherapy | 240 mg every 2 weeks, then 480 mg every 4 weeks | 358(197/161) | Placebo+chemotherapy+radiotherapy | Temozolomide+radiotherapy |  |
| Merle P, 2023[104] | HCC | Late stage | NCT02702401 | 278(226/52) | PD-1+usual care | Pembrolizumab | 200 mg | 135(112/23) | Placebo+usual care |  |  |
| Kelly RJ, 2021[105] | Esophageal cancer or gastroesophageal junction cancer | I-IV | NCT02743494 | 532(449/83) | PD-1 | Nivolumab | The dose is 240 mg, once every 2 weeks for 16 weeks, followed by 480 mg every 4 weeks | 262(222/40) | Placebo |  |  |
| Kang Y, 2022[106] | Gastric cancer | I-IV | NCT02746796 | 362(253/109) | PD-1 | Nivolumab |  | 362(270/92) | Placebo |  |  |
| Liu SV, 2021[107] | SCLC | I-IV | NCT02763579 | 201(129/72) | PD-L1+chemotherapy | Atezolizumab+Carboplatin + Etoposide | - | 202(132/70) | Placebo +chemotherapy | Carboplatin + Etoposide |  |
| Paz-Ares L, 2020[108] | NSCLC | Late stage | NCT02775435 | 278(220/58) | PD-1+chemotherapy | Pembrolizumab+carboplatin +paclitaxel/nab-paclitaxel | 200 mg | 281(235/46) | Placebo+chemotherapy | carboplatin +paclitaxel/nab-paclitaxel |  |
| Harrington KJ, 2023[109] | Head and neck squamous cell carcinoma | Late stage | NCT02823574 | 280 | CTLA-4+PD-1 | Ipilimumab+nivolumab | 1 mg/kg+3mg/kg | 143 | Placebo+PD-1 | Nivolumab |  |
| Mettu NB, 2022[110] | CRC | Late stage | NCT02873195 | 82(47/35) | PD-L1+ SMTI+ chemotherapy | Atezolizumab+ Bevacizumab+Capecitabine | 1200 mg, 7.5 mg/kg | 46(36/10) | SMTI+ chemotherapy | Bevacizumab+Capecitabine | 7.5 mg/kg |
| Kurtz JE, 2023[111] | Ovarian cancer | Late stage | NCT02891824 | 410(0/410) | PD-L1+SMTI+chemotherapy | Atezolizumab+Bevacizumab+Platinum-based | 1200mg | 204(0/204) | SMTI+chemotherapy | Bevacizumab+Platinum-based |  |
| Ascierto PA, 2022[112] | Melanoma | Late stage | NCT02908672 | 256(150/106) | PD-L1+SMTI | Atezolizumab + Cobimetinib + Vemurafenib | 840 mg | 258(149/109) | SMTI | Cobimetinib + Vemurafenib |  |
| Lee NY, 2021[113] | Head and neck squamous cell carcinoma | Late stage | NCT02952586 | 350(290/60) | PD-L1+usual care | Avelumab | 10 mg/kg | 347(285/62) | Placebo+usual care |  |  |
| Dummer R, 2022[114] | Melanoma | Late stage | NCT02967692 | 267(148/119) | PD-L1+SMTI | Spartalizumab+Dabrafenib + Trametinib | 400 mg+150mg+2mg | 265(159/106) | SMTI | Dabrafenib + Trametinib |  |
| Pal SK, 2022[115] | RCC | Late stage | NCT03024996 | 390(287/103) | PD-L1 | Atezolizumab | 1200mg | 388(278/110) | Placebo |  |  |
| Qin S, 2022[116] | HCC | Late stage | NCT03062358 | 300(257/43) | PD-1 | Pembrolizumab | 200mg | 153(126/27) | Placebo |  |  |
| Kim HR, 2023[117] | SCLC | All stage | NCT03066778 | 228(152/76) | PD-1+chemotherapy | Pembrolizumab+Etoposide+Cisplatin/Carboplatin | 200mg | 225(142/83) | Placebo+chemotherapy | Placebo+Etoposide+Cisplatin/Carboplatin |  |
| Sugawara S, 2021[118] | NSCLC | Late stage | NCT03117049 | 275(205/70) | PD-1+chemotherapy | Nivolumab+Carboplatin/paclitaxel/bevacizumab | 360mg | 275(206/89) | Placebo+chemotherapy | Carboplatin/paclitaxel/bevacizumab |  |
| Miles D, 2021[119] | BC | Late stage | NCT03125902 | 431(1/430) | PD-L1+ chemotherapy | Atezolizumab+Paclitaxel | 840 mg | 220(0/220) | Placebo+ chemotherapy | Paclitaxel |  |
| Sun J, 2021[120] | Esophagus cancer | Late stage | NCT03189719 | 373(306/67) | PD-1+usual care | Pembrolizumab | 200 mg | 376(319/57) | Placebo+usual care |  |  |
| Boyer MJ, 2021[121] | NSCLC | Late stage | NCT03302234 | 284(202/82) | PD-1+CTLA-4 | Pembrolizumab + Ipilimumab | 200mg 1mg/kg | 284(191/92) | PD-1+placebo | Pembrolizumab + Placebo |  |
| Yang Y, 2020[122] | NSCLC | Late stage | NCT03607539 | 266(204/62) | PD-1+chemotherapy | Sintilimab+Pemetrexed+Platinum | 200mg | 131(99/32) | Placebo+chemotherapy | Placebo Pemetrexed+Platinum | 200mg/m2 75mg/m2 |
| Zhou C, 2021[123] | NSCLC | Late stage | NCT03629925 | 179(163/16) | PD-1+chemotherapy | Sintilimab+Platinum+Gemcitabine | 200mg | 178(164/14) | Placebo+chemotherapy | Platinum+Gemcitabine |  |
| Zhou C, 2022[124] | NSCLC | Late stage | NCT03789604 | 320(254/66) | PD-L1+ chemotherapy | Sugemalimab+Platinum | 1200mg | 159(129/30) | Placebo+ chemotherapy | Platinum |  |
| Monk BJ, 2023[125] | Cervical cancer | Late stage | NCT03830866 | 385(0/385) | PD-L1+usual care | Durvalumab | 1500mg | 385(0/385) | placebo+usual care |  |  |
| Song Y, 2023[126] | Esophageal squamous cancer | Late stage | NCT03958890 | 368(317/51) | PD-1+chemotherapy | Serplulimab+Cisplatin +5-fluorouracil | - | 183(153/30) | Placebo+chemotherapy | Cisplatin +5-fluorouracil |  |
| Cascone T, 2024[127] | NSCLC | I-IV | NCT04025879 | 229(167/62) | PD-1 | Nivolumab | 360 mg | 232(160/72) | Usual care |  |  |
| Kirkwood JM, 2023[128] | Melanoma | IIB/C | NCT04099251 | 526(322/204) | PD-1 | Nivolumab | 480 mg | 264(161/103) | Placebo |  |  |
| Shitara K, 2024[129] | Advanced gastric or gastro-oesophageal cancer | Late stage | NCT03221426 | 402(288/114) | PD-1+chemotherapy | Pembrolizumab+5-Fluorouracil+Cisplatin/Capecitabine+Cisplatin | 200 mg | 402(287/115) | Placebo+chemotherapy | 5-Fluorouracil+Cisplatin/Capecitabine+Cisplatin |  |
| Luo H, 2021[130] | Esophageal squamous cell carcinoma | Late stage | NCT03691090 | 298(260/38) | PD-1+chemotheraoy | Camrelizumab+Paclitaxel+Cisplatin | 200mg | 298(263/35) | Placebo+chemotherapy | Paclitaxel+Cisplatin |  |
| Xu J, 2023[131] | Gastric or Gastroesophageal Junction Cancer | Late stage | NCT03745170 | 327(253/74) | PD-1+chemotherapy | Sintilimab+XELOX | Weight<60Kg：3mg/kg;>=60Kg：200 mg | 323(230/93) | Placebo+chemotherapy | XELOX |  |
| Lu Z, 2022[132] | Gastric or Gastroesophageal Junction Cancer | Late stage | NCT03748134 | 327(279/48) | PD-1+chemotherapy | Sintilimab+Cisplatin + paclitaxel /  cisplatin +5-fluorouracil | Weight<60Kg：3mg/kg;>=60Kg：200 mg | 332(288/44) | Placebo+chemotherapy | Cisplatin + paclitaxel /  cisplatin +5-fluorouracil |  |
| Lu S, 2022[133] | NSCLC | IIIb -IV | NCT03802240 | 148(61/78) | PD-1+chemotherapy | Sintilimab+Pemetrexed +cisplatin | 200mg | 145(60/85) | Placebo+chemotherapy | IBI305+Pemetrexed +cisplatin | IBI305 15 mg/kg +pemetrexed 500 mg/m²+cisplatin 75 mg/m². |
| Cheng Y, 2024[134] | SCLC | Ⅲ、Ⅳ | NCT04005716 | 227(186/41) | PD-1+chemotheraoy | Tislelizumab+Carboplatin /cisplatin | 200 mg | 230(186/44) | Placebo+chemotherapy | Carboplatin /cisplatin |  |
| Lu S, 2024[135] | NSCLC | - | NCT04158440 | 202(181/21) | PD-1+chemotheraoy | Toripalimab+Platinum-based | 4mg | 202(189/13) | Placebo+chemotherapy | Platinum-based |  |
