# Supplementary material for: Avoidance of cardiac toxicity during the application of immune checkpoint inhibitors: a safety systematic review combining network meta-analysis and pharmacovigilance study
Source: Front Immunol. 2026 Aug 31;17:1919841. doi: 10.3389/fimmu.2026.1919841 (PMC13572159; doi:10.3389/fimmu.2026.1919841)
Supplement: Supplementary file 5 [file Table5.doc]

1. André T, Elez E, Lenz H, et al. Nivolumab plus ipilimumab versus nivolumab in microsatellite instability-high metastatic colorectal cancer (CheckMate 8HW): a randomised, open-label, phase 3 trial. Lancet. 2025;405(10476):383-395. doi:10.1016/S0140-6736(24)02848-4
2. Antonia SJ, Villegas A, Daniel D, et al. Durvalumab after Chemoradiotherapy in Stage III Non-Small-Cell Lung Cancer. N Engl J Med. 2017;377(20):1919-1929. doi:10.1056/NEJMoa1709937
3. Antoniotti C, Rossini D, Pietrantonio F, et al. Upfront FOLFOXIRI plus bevacizumab with or without atezolizumab in the treatment of patients with metastatic colorectal cancer (AtezoTRIBE): a multicentre, open-label, randomised, controlled, phase 2 trial. Lancet Oncol. 2022;23(7):876-887. doi:10.1016/S1470-2045(22)00274-1
4. Barlesi F, Vansteenkiste J, Spigel D, et al. Avelumab versus docetaxel in patients with platinum-treated advanced non-small-cell lung cancer (JAVELIN Lung 200): an open-label, randomised, phase 3 study. Lancet Oncol. 2018;19(11):1468-1479. doi:10.1016/S1470-2045(18)30673-9
5. Bellmunt J, Hussain M, Gschwend JE, et al. Adjuvant atezolizumab versus observation in muscle-invasive urothelial carcinoma (IMvigor010): a multicentre, open-label, randomised, phase 3 trial. Lancet Oncol. 2021;22(4):525-537. doi:10.1016/S1470-2045(21)00004-8
6. Borghaei H, O'Byrne KJ, Paz-Ares L, et al. Nivolumab plus chemotherapy in first-line metastatic non-small-cell lung cancer: results of the phase III CheckMate 227 Part 2 trial. ESMO Open. 2023;8(6):102065. doi:10.1016/j.esmoop.2023.102065
7. Cho BC, Lee JS, Wu Y, et al. Bintrafusp Alfa Versus Pembrolizumab in Patients With Treatment-Naive, Programmed Death-Ligand 1-High Advanced NSCLC: A Randomized, Open-Label, Phase 3 Trial. J Thorac Oncol. 2023;18(12):1731-1742. doi:10.1016/j.jtho.2023.08.018
8. Choudhury AD, Kwak L, Cheung A, et al. Randomized Phase II Study Evaluating the Addition of Pembrolizumab to Radium-223 in Metastatic Castration-resistant Prostate Cancer. Cancer Immunol Res. 2024;12(6):704-718. doi:10.1158/2326-6066.CIR-22-0306
9. Choueiri TK, Penkov K, Uemura H, et al. Avelumab + axitinib versus sunitinib as first-line treatment for patients with advanced renal cell carcinoma: final analysis of the phase III JAVELIN Renal 101 trial. Ann Oncol. 2024;36(4):387-392. doi:10.1016/j.annonc.2024.12.008
10. Colombo N, Biagioli E, Harano K, et al. Atezolizumab and chemotherapy for advanced or recurrent endometrial cancer (AtTEnd): a randomised, double-blind, placebo-controlled, phase 3 trial. Lancet Oncol. 2024;25(9):1135-1146. doi:10.1016/S1470-2045(24)00334-6
11. Diaz LA, Shiu K, Kim T, et al. Pembrolizumab versus chemotherapy for microsatellite instability-high or mismatch repair-deficient metastatic colorectal cancer (KEYNOTE-177): final analysis of a randomised, open-label, phase 3 study. Lancet Oncol. 2022;23(5):659-670. doi:10.1016/S1470-2045(22)00197-8
12. Eggermont AM, Chiarion-Sileni V, Grob J. Correction to Lancet Oncol 2015; 16: 522-30. Adjuvant ipilimumab versus placebo after complete resection of high-risk stage III melanoma (EORTC 18071): a randomised, double-blind, phase 3 trial. Lancet Oncol. 2015;16(6):e262. doi:10.1016/S1470-2045(15)70271-8
13. Eggermont AM, Blank CU, Mandala M, et al. Adjuvant Pembrolizumab versus Placebo in Resected Stage III Melanoma. N Engl J Med. 2018;378(19):1789-1801. doi:10.1056/NEJMoa1802357
14. Eskander RN, Sill MW, Beffa L, et al. Pembrolizumab plus chemotherapy in advanced or recurrent endometrial cancer: overall survival and exploratory analyses of the NRG GY018 phase 3 randomized trial. Nat Med. 2025;31(5):1539-1546. doi:10.1038/s41591-025-03566-1
15. Fehrenbacher L, Spira A, Ballinger M, et al. Atezolizumab versus docetaxel for patients with previously treated non-small-cell lung cancer (POPLAR): a multicentre, open-label, phase 2 randomised controlled trial. Lancet. 2016;387(10030):1837-46. doi:10.1016/S0140-6736(16)00587-0
16. Felip E, Altorki N, Zhou C, et al. Adjuvant atezolizumab after adjuvant chemotherapy in resected stage IB-IIIA non-small-cell lung cancer (IMpower010): a randomised, multicentre, open-label, phase 3 trial. Lancet. 2021;398(10308):1344-1357. doi:10.1016/S0140-6736(21)02098-5
17. Felip E, Popat S, Dafni U, et al. A randomised phase III study of bevacizumab and carboplatin-pemetrexed chemotherapy with or without atezolizumab as first-line treatment for advanced pleural mesothelioma: results of the ETOP 13-18 BEAT-meso trial. Ann Oncol. 2025;36(5):548-560. doi:10.1016/j.annonc.2024.12.014
18. Garassino M, Gadgeel S, Speranza G, et al. Pembrolizumab Plus Pemetrexed and Platinum in Nonsquamous Non-Small-Cell Lung Cancer: 5-Year Outcomes From the Phase 3 KEYNOTE-189 Study. J Clin Oncol. 2023;41(11):1992-1998. doi:10.1200/JCO.22.01989
19. Gettinger SN, Redman MW, Bazhenova L, et al. Nivolumab Plus Ipilimumab vs Nivolumab for Previously Treated Patients With Stage IV Squamous Cell Lung Cancer: The Lung-MAP S1400I Phase 3 Randomized Clinical Trial. JAMA Oncol. 2021;7(9):1368-1377. doi:10.1001/jamaoncol.2021.2209
20. Goldman JW, Dvorkin M, Chen Y, et al. Durvalumab, with or without tremelimumab, plus platinum-etoposide versus platinum-etoposide alone in first-line treatment of extensive-stage small-cell lung cancer (CASPIAN): updated results from a randomised, controlled, open-label, phase 3 trial. Lancet Oncol. 2021;22(1):51-65. doi:10.1016/S1470-2045(20)30539-8
21. Herbst RS, Baas P, Kim D, et al. Pembrolizumab versus docetaxel for previously treated, PD-L1-positive, advanced non-small-cell lung cancer (KEYNOTE-010): a randomised controlled trial. Lancet. 2015;387(10027):1540-1550. doi:10.1016/S0140-6736(15)01281-7
22. Hodi FS, Chesney J, Pavlick AC, et al. Combined nivolumab and ipilimumab versus ipilimumab alone in patients with advanced melanoma: 2-year overall survival outcomes in a multicentre, randomised, controlled, phase 2 trial. Lancet Oncol. 2016;17(11):1558-1568. doi:10.1016/S1470-2045(16)30366-7
23. Hodi FS, Chiarion-Sileni V, Gonzalez R, et al. Nivolumab plus ipilimumab or nivolumab alone versus ipilimumab alone in advanced melanoma (CheckMate 067): 4-year outcomes of a multicentre, randomised, phase 3 trial. Lancet Oncol. 2018;19(11):1480-1492. doi:10.1016/S1470-2045(18)30700-9
24. Huang J, Xu J, Chen Y, et al. Camrelizumab versus investigator's choice of chemotherapy as second-line therapy for advanced or metastatic oesophageal squamous cell carcinoma (ESCORT): a multicentre, randomised, open-label, phase 3 study. Lancet Oncol. 2020;21(6):832-842. doi:10.1016/S1470-2045(20)30110-8
25. Janjigian YY, Kawazoe A, Yañez P, et al. The KEYNOTE-811 trial of dual PD-1 and HER2 blockade in HER2-positive gastric cancer. Nature. 2021;600(7890):727-730. doi:10.1038/s41586-021-04161-3
26. Johnson ML, Cho BC, Luft A, et al. Durvalumab With or Without Tremelimumab in Combination With Chemotherapy as First-Line Therapy for Metastatic Non-Small-Cell Lung Cancer: The Phase III POSEIDON Study. J Clin Oncol. 2022;41(6):1213-1227. doi:10.1200/JCO.22.00975
27. Kim Y, Nishio S, Kim SI, et al. Pembrolizumab plus chemotherapy with or without bevacizumab in East Asian participants with persistent, recurrent, or metastatic cervical cancer: results from KEYNOTE-826 final analysis. J Gynecol Oncol. 2025;36(4):e110. doi:10.3802/jgo.2025.36.e110
28. Kojima T, Shah MA, Muro K, et al. Randomized Phase III KEYNOTE-181 Study of Pembrolizumab Versus Chemotherapy in Advanced Esophageal Cancer. J Clin Oncol. 2020;38(35):4138-4148. doi:10.1200/JCO.20.01888
29. Langer CJ, Gadgeel SM, Borghaei H, et al. Carboplatin and pemetrexed with or without pembrolizumab for advanced, non-squamous non-small-cell lung cancer: a randomised, phase 2 cohort of the open-label KEYNOTE-021 study. Lancet Oncol. 2016;17(11):1497-1508. doi:10.1016/S1470-2045(16)30498-3
30. Lenz H, Parikh A, Spigel DR, et al. Modified FOLFOX6 plus bevacizumab with and without nivolumab for first-line treatment of metastatic colorectal cancer: phase 2 results from the CheckMate 9X8 randomized clinical trial. J Immunother Cancer. 2024;12(3). doi:10.1136/jitc-2023-008409
31. Lu S, Wang J, Yu Y, et al. Tislelizumab plus chemotherapy as first-line treatment of locally advanced or metastatic nonsquamous non-small-cell lung cancer (final analysis of RATIONALE-304: a randomized phase III trial). ESMO Open. 2024;9(10):103728. doi:10.1016/j.esmoop.2024.103728
32. Luke JJ, Ascierto PA, Khattak MA, et al. Pembrolizumab Versus Placebo as Adjuvant Therapy in Resected Stage IIB or IIC Melanoma: Final Analysis of Distant Metastasis-Free Survival in the Phase III KEYNOTE-716 Study. J Clin Oncol. 2024;42(14):1619-1624. doi:10.1200/JCO.23.02355
33. Machiels J, Tao Y, Licitra L, et al. Pembrolizumab plus concurrent chemoradiotherapy versus placebo plus concurrent chemoradiotherapy in patients with locally advanced squamous cell carcinoma of the head and neck (KEYNOTE-412): a randomised, double-blind, phase 3 trial. Lancet Oncol. 2024;25(5):572-587. doi:10.1016/S1470-2045(24)00100-1
34. Mai H, Chen Q, Chen D, et al. Publisher Correction: Toripalimab or placebo plus chemotherapy as first-line treatment in advanced nasopharyngeal carcinoma: a multicenter randomized phase 3 trial. Nat Med. 2022;28(1):214. doi:10.1038/s41591-021-01673-3
35. Maio M, Scherpereel A, Calabrò L, et al. Tremelimumab as second-line or third-line treatment in relapsed malignant mesothelioma (DETERMINE): a multicentre, international, randomised, double-blind, placebo-controlled phase 2b trial. Lancet Oncol. 2017;18(9):1261-1273. doi:10.1016/S1470-2045(17)30446-1
36. Mell LK, Torres-Saavedra PA, Wong SJ, et al. Radiotherapy with cetuximab or durvalumab for locoregionally advanced head and neck cancer in patients with a contraindication to cisplatin (NRG-HN004): an open-label, multicentre, parallel-group, randomised, phase 2/3 trial. Lancet Oncol. 2024;25(12):1576-1588. doi:10.1016/S1470-2045(24)00507-2
37. Mok TS, Wu Y, Kudaba I, et al. Pembrolizumab versus chemotherapy for previously untreated, PD-L1-expressing, locally advanced or metastatic non-small-cell lung cancer (KEYNOTE-042): a randomised, open-label, controlled, phase 3 trial. Lancet. 2019;393(10183):1819-1830. doi:10.1016/S0140-6736(18)32409-7
38. Monk BJ, Colombo N, Oza AM, et al. Chemotherapy with or without avelumab followed by avelumab maintenance versus chemotherapy alone in patients with previously untreated epithelial ovarian cancer (JAVELIN Ovarian 100): an open-label, randomised, phase 3 trial. Lancet Oncol. 2021;22(9):1275-1289. doi:10.1016/S1470-2045(21)00342-9
39. Moore KN, Bookman M, Sehouli J, et al. Atezolizumab, Bevacizumab, and Chemotherapy for Newly Diagnosed Stage III or IV Ovarian Cancer: Placebo-Controlled Randomized Phase III Trial (IMagyn050/GOG 3015/ENGOT-OV39). J Clin Oncol. 2021;39(17):1842-1855. doi:10.1200/JCO.21.00306
40. Motzer RJ, Rini BI, McDermott DF, et al. Nivolumab plus ipilimumab versus sunitinib in first-line treatment for advanced renal cell carcinoma: extended follow-up of efficacy and safety results from a randomised, controlled, phase 3 trial. Lancet Oncol. 2019;20(10):1370-1385. doi:10.1016/S1470-2045(19)30413-9
41. Felip E, Altorki NK, Zhou C, et al. Five-Year Survival Outcomes With Atezolizumab After Chemotherapy in Resected Stage IB-IIIA Non-Small Cell Lung Cancer (IMpower010): An Open-Label, Randomized, Phase III Trial. J Clin Oncol. 2025;43(21):2343-2349. doi:10.1200/JCO-24-01681
42. Zhou A, Shen P, Li J, et al. First-line benmelstobart plus anlotinib versus sunitinib in advanced renal cell carcinoma (ETER100): a multicentre, randomised, open-label, phase 3 trial. Lancet Oncol. 2025;26(9):1145-1157. doi:10.1016/S1470-2045(25)00343-2
43. Zeidan AM, Boss I, Beach CL, et al. A randomized phase 2 trial of azacitidine with or without durvalumab as first-line therapy for older patients with AML. Blood Adv. 2022;6(7):2219-2229. doi:10.1182/bloodadvances.2021006138
44. Baas P, Scherpereel A, Nowak AK, et al. First-line nivolumab plus ipilimumab in unresectable malignant pleural mesothelioma (CheckMate 743): a multicentre, randomised, open-label, phase 3 trial. Lancet. 2021;397(10272):375-386. doi:10.1016/S0140-6736(20)32714-8
45. Yang JC, Lee DH, Lee J, et al. Phase III KEYNOTE-789 Study of Pemetrexed and Platinum With or Without Pembrolizumab for Tyrosine Kinase Inhibitor‒Resistant, EGFR-Mutant, Metastatic Nonsquamous Non-Small Cell Lung Cancer. J Clin Oncol. 2024;42(34):4029-4039. doi:10.1200/JCO.23.02747
46. Xu J, Kato K, Raymond E, et al. Tislelizumab plus chemotherapy versus placebo plus chemotherapy as first-line treatment for advanced or metastatic oesophageal squamous cell carcinoma (RATIONALE-306): a global, randomised, placebo-controlled, phase 3 study. Lancet Oncol. 2023;24(5):483-495. doi:10.1016/S1470-2045(23)00108-0
47. Xing W, Zhao L, Zheng Y, et al. The Sequence of Chemotherapy and Toripalimab Might Influence the Efficacy of Neoadjuvant Chemoimmunotherapy in Locally Advanced Esophageal Squamous Cell Cancer-A Phase II Study. Front Immunol. 2021;12:772450. doi:10.3389/fimmu.2021.772450
48. Xiao W, Chen G, Gao Y, et al. Effect of neoadjuvant chemoradiotherapy with or without PD-1 antibody sintilimab in pMMR locally advanced rectal cancer: A randomized clinical trial. Cancer Cell. 2024;42(9):1570-1581.e4. doi:10.1016/j.ccell.2024.07.004
49. Winer EP, Lipatov O, Im S, et al. Pembrolizumab versus investigator-choice chemotherapy for metastatic triple-negative breast cancer (KEYNOTE-119): a randomised, open-label, phase 3 trial. Lancet Oncol. 2021;22(4):499-511. doi:10.1016/S1470-2045(20)30754-3
50. West H, McCleod M, Hussein M, et al. Atezolizumab in combination with carboplatin plus nab-paclitaxel chemotherapy compared with chemotherapy alone as first-line treatment for metastatic non-squamous non-small-cell lung cancer (IMpower130): a multicentre, randomised, open-label, phase 3 trial. Lancet Oncol. 2019;20(7):924-937. doi:10.1016/S1470-2045(19)30167-6
51. Vano Y, Elaidi R, Bennamoun M, et al. Nivolumab, nivolumab-ipilimumab, and VEGFR-tyrosine kinase inhibitors as first-line treatment for metastatic clear-cell renal cell carcinoma (BIONIKK): a biomarker-driven, open-label, non-comparative, randomised, phase 2 trial. Lancet Oncol. 2022;23(5):612-624. doi:10.1016/S1470-2045(22)00128-0
52. Usmani SZ, Schjesvold F, Oriol A, et al. Pembrolizumab plus lenalidomide and dexamethasone for patients with treatment-naive multiple myeloma (KEYNOTE-185): a randomised, open-label, phase 3 trial. Lancet Haematol. 2019;6(9):e448-e458. doi:10.1016/S2352-3026(19)30109-7
53. Tian F, Dai H, Sha D, et al. Total neoadjuvant treatment with short-course radiotherapy followed by sintilimab plus capecitabine-oxaliplatin versus short-course radiotherapy followed by capecitabine-oxaliplatin in patients with locally advanced rectal cancer (SPRING-01): a single-centre, open-label, phase 2, randomised controlled trial. Lancet Oncol. 2025;26(8):1043-1054. doi:10.1016/S1470-2045(25)00286-4
54. Takahashi M, Cortes J, Dent R, et al. Pembrolizumab Plus Chemotherapy Followed by Pembrolizumab in Patients With Early Triple-Negative Breast Cancer: A Secondary Analysis of a Randomized Clinical Trial. JAMA Netw Open. 2023;6(11):e2342107. doi:10.1001/jamanetworkopen.2023.42107
55. Sun L, Gong Z, Wang L, et al. Multi-center phase II study of nab-paclitaxel plus camrelizumab versus nab-paclitaxel alone as second-line treatment for advanced gastric cancer. Oncologist. 2025;30(7). doi:10.1093/oncolo/oyaf189
56. Spicer JD, Garassino MC, Wakelee H, et al. Neoadjuvant pembrolizumab plus chemotherapy followed by adjuvant pembrolizumab compared with neoadjuvant chemotherapy alone in patients with early-stage non-small-cell lung cancer (KEYNOTE-671): a randomised, double-blind, placebo-controlled, phase 3 trial. Lancet. 2024;404(10459):1240-1252. doi:10.1016/S0140-6736(24)01756-2
57. Sezer A, Kilickap S, Gümüş M, et al. Cemiplimab monotherapy for first-line treatment of advanced non-small-cell lung cancer with PD-L1 of at least 50%: a multicentre, open-label, global, phase 3, randomised, controlled trial. Lancet. 2021;397(10274):592-604. doi:10.1016/S0140-6736(21)00228-2
58. Scherpereel A, Mazieres J, Greillier L, et al. Nivolumab or nivolumab plus ipilimumab in patients with relapsed malignant pleural mesothelioma (IFCT-1501 MAPS2): a multicentre, open-label, randomised, non-comparative, phase 2 trial. Lancet Oncol. 2019;20(2):239-253. doi:10.1016/S1470-2045(18)30765-4
59. Rubinstein MM, Doria ER, Konner J, et al. Durvalumab with or without tremelimumab in patients with persistent or recurrent endometrial cancer or endometrial carcinosarcoma: A randomized open-label phase 2 study. Gynecol Oncol. 2022;169:64-69. doi:10.1016/j.ygyno.2022.11.028
60. Rini BI, Moslehi JJ, Bonaca M, et al. Prospective Cardiovascular Surveillance of Immune Checkpoint Inhibitor-Based Combination Therapy in Patients With Advanced Renal Cell Cancer: Data From the Phase III JAVELIN Renal 101 Trial. J Clin Oncol. 2022;40(17):1929-1938. doi:10.1200/JCO.21.01806
61. Ribas A, Kefford R, Marshall MA, et al. Phase III randomized clinical trial comparing tremelimumab with standard-of-care chemotherapy in patients with advanced melanoma. J Clin Oncol. 2013;31(5):616-22. doi:10.1200/JCO.2012.44.6112
62. Rha SY, Oh D, Yañez P, et al. Pembrolizumab plus chemotherapy versus placebo plus chemotherapy for HER2-negative advanced gastric cancer (KEYNOTE-859): a multicentre, randomised, double-blind, phase 3 trial. Lancet Oncol. 2023;24(11):1181-1195. doi:10.1016/S1470-2045(23)00515-6
63. Reckamp KL, Redman MW, Dragnev KH, et al. Phase II Randomized Study of Ramucirumab and Pembrolizumab Versus Standard of Care in Advanced Non-Small-Cell Lung Cancer Previously Treated With Immunotherapy-Lung-MAP S1800A. J Clin Oncol. 2022;40(21):2295-2306. doi:10.1200/JCO.22.00912
64. Özgüroğlu M, Kilickap S, Sezer A, et al. First-line cemiplimab monotherapy and continued cemiplimab beyond progression plus chemotherapy for advanced non-small-cell lung cancer with PD-L1 50% or more (EMPOWER-Lung 1): 35-month follow-up from a mutlicentre, open-label, randomised, phase 3 trial. Lancet Oncol. 2023;24(9):989-1001. doi:10.1016/S1470-2045(23)00329-7
65. Paz-Ares L, Marreaud S, Dafni U, et al. Pembrolizumab versus placebo as adjuvant therapy for completely resected stage IB-IIIA non-small-cell lung cancer (PEARLS/KEYNOTE-091): an interim analysis of a randomised, triple-blind, phase 3 trial. Lancet Oncol. 2022;23(10):1274-1286. doi:10.1016/S1470-2045(22)00518-6
66. Nishio M, Barlesi F, West H, et al. Atezolizumab Plus Chemotherapy for First-Line Treatment of Nonsquamous NSCLC: Results From the Randomized Phase 3 IMpower132 Trial. J Thorac Oncol. 2020;16(4):653-664. doi:10.1016/j.jtho.2020.11.025
67. Powles T, Tomczak P, Park SH, et al. Pembrolizumab versus placebo as post-nephrectomy adjuvant therapy for clear cell renal cell carcinoma (KEYNOTE-564): 30-month follow-up analysis of a multicentre, randomised, double-blind, placebo-controlled, phase 3 trial. Lancet Oncol. 2022;23(9):1133-1144. doi:10.1016/S1470-2045(22)00487-9
68. Powles T, Plimack ER, Soulières D, et al. Pembrolizumab plus axitinib versus sunitinib monotherapy as first-line treatment of advanced renal cell carcinoma (KEYNOTE-426): extended follow-up from a randomised, open-label, phase 3 trial. Lancet Oncol. 2020;21(12):1563-1573. doi:10.1016/S1470-2045(20)30436-8
69. Pignata S, Scambia G, Schettino C, et al. Carboplatin and paclitaxel plus avelumab compared with carboplatin and paclitaxel in advanced or recurrent endometrial cancer (MITO END-3): a multicentre, open-label, randomised, controlled, phase 2 trial. Lancet Oncol. 2023;24(3):286-296. doi:10.1016/S1470-2045(23)00016-5
70. Paz-Ares L, Borghaei H, Liu SV, et al. Efficacy and safety of first-line maintenance therapy with lurbinectedin plus atezolizumab in extensive-stage small-cell lung cancer (IMforte): a randomised, multicentre, open-label, phase 3 trial. Lancet. 2025;405(10495):2129-2143. doi:10.1016/S0140-6736(25)01011-6
71. Long GV, Carlino MS, McNeil C, et al. Pembrolizumab versus ipilimumab for advanced melanoma: 10-year follow-up of the phase III KEYNOTE-006 study. Ann Oncol. 2024;35(12):1191-1199. doi:10.1016/j.annonc.2024.08.2330
72. Palbociclib After CDK and Endocrine Therapy (PACE) https://clinicaltrials.gov/study/NCT03147287?cond=Palbociclib%20After%20CDK%20and%20Endocrine%20Therapy%20(PACE)%20&rank=1
73. Phase 3 Study of Sitravatinib Plus Nivolumab vs Docetaxel in Patients With Advanced Non-Squamous Non-Small Cell Lung Cancer (SAPPHIRE)https://clinicaltrials.gov/study/NCT03906071?cond=%20SAPPHIRE&page=1&rank=5&tab=table
74. Nivolumab in Treating Patients With Localized Kidney Cancer Undergoing Nephrectomy.https://clinicaltrials.gov/study/NCT03055013?cond=Nivolumab%20in%20Treating%20Patients%20With%20Localized%20Kidney%20Cancer%20Undergoing%20Nephrectomy&rank=1
75. Ascierto PA, Ferrucci PF, Fisher R, et al. Dabrafenib, trametinib and pembrolizumab or placebo in BRAF-mutant melanoma. Nat Med. 2019;25(6):941-946. doi:10.1038/s41591-019-0448-9
76. Bang Y, Cho JY, Kim YH, et al. Efficacy of Sequential Ipilimumab Monotherapy versus Best Supportive Care for Unresectable Locally Advanced/Metastatic Gastric or Gastroesophageal Junction Cancer. Clin Cancer Res. 2017;23(19):5671-5678. doi:10.1158/1078-0432.CCR-17-0025
77. Cella D, Grünwald V, Escudier B, et al. Patient-reported outcomes of patients with advanced renal cell carcinoma treated with nivolumab plus ipilimumab versus sunitinib (CheckMate 214): a randomised, phase 3 trial. Lancet Oncol. 2019;20(2):297-310. doi:10.1016/S1470-2045(18)30778-2
78. Motzer RJ, Escudier B, George S, et al. Nivolumab versus everolimus in patients with advanced renal cell carcinoma: Updated results with long-term follow-up of the randomized, open-label, phase 3 CheckMate 025 trial. Cancer. 2020;126(18):4156-4167. doi:10.1002/cncr.33033
79. Cohen EE, Soulières D, Le Tourneau C, et al. Pembrolizumab versus methotrexate, docetaxel, or cetuximab for recurrent or metastatic head-and-neck squamous cell carcinoma (KEYNOTE-040): a randomised, open-label, phase 3 study. Lancet. 2018;393(10167):156-167. doi:10.1016/S0140-6736(18)31999-8
80. Cortes J, Cescon DW, Rugo HS, et al. Pembrolizumab plus chemotherapy versus placebo plus chemotherapy for previously untreated locally recurrent inoperable or metastatic triple-negative breast cancer (KEYNOTE-355): a randomised, placebo-controlled, double-blind, phase 3 clinical trial. Lancet. 2020;396(10265):1817-1828. doi:10.1016/S0140-6736(20)32531-9
81. Emens LA, Esteva FJ, Beresford M, et al. Trastuzumab emtansine plus atezolizumab versus trastuzumab emtansine plus placebo in previously treated, HER2-positive advanced breast cancer (KATE2): a phase 2, multicentre, randomised, double-blind trial. Lancet Oncol. 2020;21(10):1283-1295. doi:10.1016/S1470-2045(20)30465-4
82. An Investigational Study of Immunotherapy Combinations With Chemotherapy in Patients With Gastric or Gastroesophageal Junction (GEJ) Cancers. https://clinicaltrials.gov/study/NCT03662659?cond=An%20Investigational%20Study%20of%20Immunotherapy%20Combinations%20With%20Chemotherapy%20in%20Patients%20With%20Gastric%20or%20Gastroesophageal%20Junction%20(GEJ)%20Cancers&rank=1
83. Kato K, Cho BC, Takahashi M, et al. Nivolumab versus chemotherapy in patients with advanced oesophageal squamous cell carcinoma refractory or intolerant to previous chemotherapy (ATTRACTION-3): a multicentre, randomised, open-label, phase 3 trial. Lancet Oncol. 2019;20(11):1506-1517. doi:10.1016/S1470-2045(19)30626-6
84. Llovet JM, Kudo M, Merle P, et al. Lenvatinib plus pembrolizumab versus lenvatinib plus placebo for advanced hepatocellular carcinoma (LEAP-002): a randomised, double-blind, phase 3 trial. Lancet Oncol. 2023;24(12):1399-1410. doi:10.1016/S1470-2045(23)00469-2
85. A Phase 3 Study of Pembrolizumab + Epacadostat or Placebo in Subjects With Unresectable or Metastatic Melanoma (Keynote-252 / ECHO-301) ECHO-301)https://clinicaltrials.gov/study/NCT02752074?cond=NCT02752074&rank=1.
86. Marth C, Moore RG, Bidziński M, et al. First-Line Lenvatinib Plus Pembrolizumab Versus Chemotherapy for Advanced Endometrial Cancer: A Randomized, Open-Label, Phase III Trial. J Clin Oncol. 2024;43(9):1083-1100. doi:10.1200/JCO-24-01326
87. Monk BJ, Colombo N, Oza AM, et al. Chemotherapy with or without avelumab followed by avelumab maintenance versus chemotherapy alone in patients with previously untreated epithelial ovarian cancer (JAVELIN Ovarian 100): an open-label, randomised, phase 3 trial. Lancet Oncol. 2021;22(9):1275-1289. doi:10.1016/S1470-2045(21)00342-9
88. Motzer RJ, Powles T, Burotto M, et al. Nivolumab plus cabozantinib versus sunitinib in first-line treatment for advanced renal cell carcinoma (CheckMate 9ER): long-term follow-up results from an open-label, randomised, phase 3 trial. Lancet Oncol. 2022;23(7):888-898. doi:10.1016/S1470-2045(22)00290-X
89. Eng C, Kim TW, Bendell J, et al. Atezolizumab with or without cobimetinib versus regorafenib in previously treated metastatic colorectal cancer (IMblaze370): a multicentre, open-label, phase 3, randomised, controlled trial. Lancet Oncol. 2019;20(6):849-861. doi:10.1016/S1470-2045(19)30027-0
90. Hodi FS, McDermott DF, Weber RW, et al. Improved survival with ipilimumab in patients with metastatic melanoma. N Engl J Med. 2010;363(8):711-23. doi:10.1056/NEJMoa1003466
91. Robert C, Thomas L, Bondarenko I, et al. Ipilimumab plus dacarbazine for previously untreated metastatic melanoma. N Engl J Med. 2011;364(26):2517-26. doi:10.1056/NEJMoa1104621
92. Reck M, Bondarenko I, Luft A, et al. Ipilimumab in combination with paclitaxel and carboplatin as first-line therapy in extensive-disease-small-cell lung cancer: results from a randomized, double-blind, multicenter phase 2 trial. Ann Oncol. 2012;24(1):75-83. doi:10.1093/annonc/mds213
93. Lynch TJ, Bondarenko I, Luft A, et al. Ipilimumab in combination with paclitaxel and carboplatin as first-line treatment in stage IIIB/IV non-small-cell lung cancer: results from a randomized, double-blind, multicenter phase II study. J Clin Oncol. 2012;30(17):2046-54. doi:10.1200/JCO.2011.38.4032
94. Kwon ED, Drake CG, Scher HI, et al. Ipilimumab versus placebo after radiotherapy in patients with metastatic castration-resistant prostate cancer that had progressed after docetaxel chemotherapy (CA184-043): a multicentre, randomised, double-blind, phase 3 trial. Lancet Oncol. 2014;15(7):700-12. doi:10.1016/S1470-2045(14)70189-5
95. Beer TM, Kwon ED, Drake CG, et al. Randomized, Double-Blind, Phase III Trial of Ipilimumab Versus Placebo in Asymptomatic or Minimally Symptomatic Patients With Metastatic Chemotherapy-Naive Castration-Resistant Prostate Cancer. J Clin Oncol. 2016;35(1):40-47. doi:10.1200/JCO.2016.69.1584
96. Govindan R, Szczęsna A, Ahn M, et al. Phase III Trial of Ipilimumab Combined With Paclitaxel and Carboplatin in Advanced Squamous Non-Small-Cell Lung Cancer. J Clin Oncol. 2017;35(30):3449-3457. doi:10.1200/JCO.2016.71.7629
97. Kang Y, Boku N, Satoh T, et al. Nivolumab in patients with advanced gastric or gastro-oesophageal junction cancer refractory to, or intolerant of, at least two previous chemotherapy regimens (ONO-4538-12, ATTRACTION-2): a randomised, double-blind, placebo-controlled, phase 3 trial. Lancet. 2017;390(10111):2461-2471. doi:10.1016/S0140-6736(17)31827-5
98. Emens LA, Adams S, Barrios CH, et al. Corrigendum to 'First-line atezolizumab plus nab-paclitaxel for unresectable locally advanced or metastatic triple-negative breast cancer: IMpassion130 final overall survival analysis': Annals of Oncology 2021; volume 32: 983-993. Ann Oncol. 2021;32(10):1308. doi:10.1016/j.annonc.2021.07.013
99. Shitara K, Van Cutsem E, Bang Y, et al. Efficacy and Safety of Pembrolizumab or Pembrolizumab Plus Chemotherapy vs Chemotherapy Alone for Patients With First-line, Advanced Gastric Cancer: The KEYNOTE-062 Phase 3 Randomized Clinical Trial. JAMA Oncol. 2020;6(10):1571-1580. doi:10.1001/jamaoncol.2020.3370
100. Galsky MD, Mortazavi A, Milowsky MI, et al. Randomized Double-Blind Phase II Study of Maintenance Pembrolizumab Versus Placebo After First-Line Chemotherapy in Patients With Metastatic Urothelial Cancer. J Clin Oncol. 2020;38(16):1797-1806. doi:10.1200/JCO.19.03091
101. Owonikoko TK, Park K, Govindan R, et al. Nivolumab and Ipilimumab as Maintenance Therapy in Extensive-Disease Small-Cell Lung Cancer: CheckMate 451. J Clin Oncol. 2021;39(12):1349-1359. doi:10.1200/JCO.20.02212
102. Bajorin DF, Witjes JA, Gschwend JE, et al. Adjuvant Nivolumab versus Placebo in Muscle-Invasive Urothelial Carcinoma. N Engl J Med. 2021;384(22):2102-2114. doi:10.1056/NEJMoa2034442
103. Lim M, Weller M, Idbaih A, et al. Phase III trial of chemoradiotherapy with temozolomide plus nivolumab or placebo for newly diagnosed glioblastoma with methylated MGMT promoter. Neuro-oncology. 2022;24(11):1935-1949. doi:10.1093/neuonc/noac116
104. Merle P, Kudo M, Edeline J, Bouattour M, Cheng AL, Chan SL, Yau T, Garrido M, Knox J, Daniele B, Breder V, Lim HY, Ogasawara S, Cattan S, Chao Y, Siegel AB, Martinez-Forero I, Wei Z, Liu CC, Finn RS. Pembrolizumab as Second-Line Therapy for Advanced Hepatocellular Carcinoma: Longer Term Follow-Up from the Phase 3 KEYNOTE-240 Trial. Liver Cancer. 2023 Feb 14;12(4):309-320. doi: 10.1159/000529636. PMID: 37901200; PMCID: PMC10601873.
105. Kelly RJ, Ajani JA, Kuzdzal J, et al. Adjuvant Nivolumab in Resected Esophageal or Gastroesophageal Junction Cancer. N Engl J Med. 2021;384(13):1191-1203. doi:10.1056/NEJMoa2032125
106. Kang Y, Chen L, Ryu M, et al. Nivolumab plus chemotherapy versus placebo plus chemotherapy in patients with HER2-negative, untreated, unresectable advanced or recurrent gastric or gastro-oesophageal junction cancer (ATTRACTION-4): a randomised, multicentre, double-blind, placebo-controlled, phase 3 trial. Lancet Oncol. 2022;23(2):234-247. doi:10.1016/S1470-2045(21)00692-6
107. Liu SV, Reck M, Mansfield AS, et al. Updated Overall Survival and PD-L1 Subgroup Analysis of Patients With Extensive-Stage Small-Cell Lung Cancer Treated With Atezolizumab, Carboplatin, and Etoposide (IMpower133). J Clin Oncol. 2021;39(6):619-630. doi:10.1200/JCO.20.01055
108. Paz-Ares L, Vicente D, Tafreshi A, et al. A Randomized, Placebo-Controlled Trial of Pembrolizumab Plus Chemotherapy in Patients With Metastatic Squamous NSCLC: Protocol-Specified Final Analysis of KEYNOTE-407. J Thorac Oncol. 2020;15(10):1657-1669. doi:10.1016/j.jtho.2020.06.015
109. Harrington KJ, Ferris RL, Gillison M, et al. Efficacy and Safety of Nivolumab Plus Ipilimumab vs Nivolumab Alone for Treatment of Recurrent or Metastatic Squamous Cell Carcinoma of the Head and Neck: The Phase 2 CheckMate 714 Randomized Clinical Trial. JAMA Oncol. 2023;9(6):779-789. doi:10.1001/jamaoncol.2023.0147
110. Mettu NB, Ou F, Zemla TJ, et al. Assessment of Capecitabine and Bevacizumab With or Without Atezolizumab for the Treatment of Refractory Metastatic Colorectal Cancer: A Randomized Clinical Trial. JAMA Netw Open. 2022;5(2):e2149040. doi:10.1001/jamanetworkopen.2021.49040
111. Kurtz JE, Pujade-Lauraine E, Oaknin A, Belin L, Leitner K, Cibula D, Denys H, Rosengarten O, Rodrigues M, de Gregorio N, Martinez García J, Petru E, Kocián R, Vergote I, Pautier P, Schmalfeldt B, Gaba L, Polterauer S, Mouret Reynier MA, Sehouli J, Churruca C, Selle F, Joly F, D'Hondt V, Bultot-Boissier É, Lebreton C, Lotz JP, Largillier R, Heudel PE, Heitz F; ATALANTE/ENGOT-ov29 Investigators. Atezolizumab Combined With Bevacizumab and Platinum-Based Therapy for Platinum-Sensitive Ovarian Cancer: Placebo-Controlled Randomized Phase III ATALANTE/ENGOT-ov29 Trial. J Clin Oncol. 2023 Oct 20;41(30):4768-4778. doi: 10.1200/JCO.23.00529. Epub 2023 Aug 29. PMID: 37643382; PMCID: PMC10602539.
112. Ascierto PA, Stroyakovskiy D, Gogas H, et al. Overall survival with first-line atezolizumab in combination with vemurafenib and cobimetinib in BRAFV600 mutation-positive advanced melanoma (IMspire150): second interim analysis of a multicentre, randomised, phase 3 study. Lancet Oncol. 2022;24(1):33-44. doi:10.1016/S1470-2045(22)00687-8
113. Lee NY, Ferris RL, Psyrri A, et al. Avelumab plus standard-of-care chemoradiotherapy versus chemoradiotherapy alone in patients with locally advanced squamous cell carcinoma of the head and neck: a randomised, double-blind, placebo-controlled, multicentre, phase 3 trial. Lancet Oncol. 2021;22(4):450-462. doi:10.1016/S1470-2045(20)30737-3
114. Dummer R, Long GV, Robert C, Tawbi HA, Flaherty KT, Ascierto PA, Nathan PD, Rutkowski P, Leonov O, Dutriaux C, Mandalà M, Lorigan P, Ferrucci PF, Grob JJ, Meyer N, Gogas H, Stroyakovskiy D, Arance A, Brase JC, Green S, Haas T, Masood A, Gasal E, Ribas A, Schadendorf D. Randomized Phase III Trial Evaluating Spartalizumab Plus Dabrafenib and Trametinib for BRAF V600-Mutant Unresectable or Metastatic Melanoma. J Clin Oncol. 2022 May 1;40(13):1428-1438. doi: 10.1200/JCO.21.01601. Epub 2022 Jan 14. PMID: 35030011; PMCID: PMC9061149.
115. Pal SK, Uzzo R, Karam JA, et al. Adjuvant atezolizumab versus placebo for patients with renal cell carcinoma at increased risk of recurrence following resection (IMmotion010): a multicentre, randomised, double-blind, phase 3 trial. Lancet. 2022;400(10358):1103-1116. doi:10.1016/S0140-6736(22)01658-0
116. Qin S, Chen Z, Fang W, et al. Pembrolizumab Versus Placebo as Second-Line Therapy in Patients From Asia With Advanced Hepatocellular Carcinoma: A Randomized, Double-Blind, Phase III Trial. J Clin Oncol. 2022;41(7):1434-1443. doi:10.1200/JCO.22.00620
117. Kim HR, Awad MM, Navarro A, et al. Patient-Reported Health-Related Quality of Life in KEYNOTE-604: Pembrolizumab or Placebo Added to Etoposide and Platinum as First-Line Therapy for Extensive-Stage SCLC. JTO Clin Res Rep 2023;4(11):100572. doi:10.1016/j.jtocrr.2023.100572
118. Sugawara S, Lee J, Kang J, et al. Nivolumab with carboplatin, paclitaxel, and bevacizumab for first-line treatment of advanced nonsquamous non-small-cell lung cancer. Ann Oncol. 2021;32(9):1137-1147. doi:10.1016/j.annonc.2021.06.004
119. Miles D, Gligorov J, André F, et al. Primary results from IMpassion131, a double-blind, placebo-controlled, randomised phase III trial of first-line paclitaxel with or without atezolizumab for unresectable locally advanced/metastatic triple-negative breast cancer. Ann Oncol. 2021;32(8):994-1004. doi:10.1016/j.annonc.2021.05.801
120. Sun J, Shen L, Shah MA, et al. Pembrolizumab plus chemotherapy versus chemotherapy alone for first-line treatment of advanced oesophageal cancer (KEYNOTE-590): a randomised, placebo-controlled, phase 3 study. Lancet. 2021;398(10302):759-771. doi:10.1016/S0140-6736(21)01234-4
121. Boyer MJ, Şendur MA, Rodríguez-Abreu D, et al. Pembrolizumab Plus Ipilimumab or Placebo for Metastatic Non-Small-Cell Lung Cancer With PD-L1 Tumor Proportion Score ≥ 50%: Randomized, Double-Blind Phase III KEYNOTE-598 Study. J Clin Oncol. 2021;39(21):2327-2338. doi:10.1200/JCO.20.03579
122. Yang Y, Wang Z, Fang J, et al. Efficacy and Safety of Sintilimab Plus Pemetrexed and Platinum as First-Line Treatment for Locally Advanced or Metastatic Nonsquamous NSCLC: a Randomized, Double-Blind, Phase 3 Study (Oncology pRogram by InnovENT anti-PD-1-11). J Thorac Oncol. 2020;15(10):1636-1646. doi:10.1016/j.jtho.2020.07.014
123. Zhou C, Wu L, Fan Y, et al. Sintilimab Plus Platinum and Gemcitabine as First-Line Treatment for Advanced or Metastatic Squamous NSCLC: Results From a Randomized, Double-Blind, Phase 3 Trial (ORIENT-12). J Thorac Oncol. 2021;16(9):1501-1511. doi:10.1016/j.jtho.2021.04.011
124. Zhou C, Wang Z, Sun Y, et al. Sugemalimab versus placebo, in combination with platinum-based chemotherapy, as first-line treatment of metastatic non-small-cell lung cancer (GEMSTONE-302): interim and final analyses of a double-blind, randomised, phase 3 clinical trial. Lancet Oncol. 2022;23(2):220-233. doi:10.1016/S1470-2045(21)00650-1
125. Monk BJ, Toita T, Wu X, et al. Durvalumab versus placebo with chemoradiotherapy for locally advanced cervical cancer (CALLA): a randomised, double-blind, phase 3 trial. Lancet Oncol. 2023;24(12):1334-1348. doi:10.1016/S1470-2045(23)00479-5
126. Song Y, Zhang B, Xin D, et al. First-line serplulimab or placebo plus chemotherapy in PD-L1-positive esophageal squamous cell carcinoma: a randomized, double-blind phase 3 trial. Nat Med. 2023;29(2):473-482. doi:10.1038/s41591-022-02179-2
127. Cascone T, Awad MM, Spicer JD, He J, Lu S, Sepesi B, Tanaka F, Taube JM, Cornelissen R, Havel L, Karaseva N, Kuzdzal J, Petruzelka LB, Wu L, Pujol JL, Ito H, Ciuleanu TE, de Oliveira Muniz Koch L, Janssens A, Alexandru A, Bohnet S, Moiseyenko FV, Gao Y, Watanabe Y, Coronado Erdmann C, Sathyanarayana P, Meadows-Shropshire S, Blum SI, Provencio Pulla M; CheckMate 77T Investigators. Perioperative Nivolumab in Resectable Lung Cancer. N Engl J Med. 2024 May 16;390(19):1756-1769. doi: 10.1056/NEJMoa2311926. PMID: 38749033.
128. Kirkwood JM, Del Vecchio M, Weber J, et al. Adjuvant nivolumab in resected stage IIB/C melanoma: primary results from the randomized, phase 3 CheckMate 76K trial. Nat Med. 2023;29(11):2835-2843. doi:10.1038/s41591-023-02583-2
129. Shitara K, Rha SY, Wyrwicz LS, Oshima T, Karaseva N, Osipov M, Yasui H, Yabusaki H, Afanasyev S, Park YK, Al-Batran SE, Yoshikawa T, Yanez P, Dib Bartolomeo M, Lonardi S, Tabernero J, Van Cutsem E, Janjigian YY, Oh DY, Xu J, Fang X, Shih CS, Bhagia P, Bang YJ; KEYNOTE-585 investigators. Neoadjuvant and adjuvant pembrolizumab plus chemotherapy in locally advanced gastric or gastro-oesophageal cancer (KEYNOTE-585): an interim analysis of the multicentre, double-blind, randomised phase 3 study. Lancet Oncol. 2024 Feb;25(2):212-224. doi: 10.1016/S1470-2045(23)00541-7. Epub 2023 Dec 19. PMID: 38134948.
130. Luo H, Lu J, Bai Y, Mao T, Wang J, Fan Q, Zhang Y, Zhao K, Chen Z, Gao S, Li J, Fu Z, Gu K, Liu Z, Wu L, Zhang X, Feng J, Niu Z, Ba Y, Zhang H, Liu Y, Zhang L, Min X, Huang J, Cheng Y, Wang D, Shen Y, Yang Q, Zou J, Xu RH; ESCORT-1st Investigators. Effect of Camrelizumab vs Placebo Added to Chemotherapy on Survival and Progression-Free Survival in Patients With Advanced or Metastatic Esophageal Squamous Cell Carcinoma: The ESCORT-1st Randomized Clinical Trial. JAMA. 2021 Sep 14;326(10):916-925. doi: 10.1001/jama.2021.12836. PMID: 34519801; PMCID: PMC8441593.
131. Xu J, Jiang H, Pan Y, et al. Sintilimab Plus Chemotherapy for Unresectable Gastric or Gastroesophageal Junction Cancer: The ORIENT-16 Randomized Clinical Trial. JAMA. 2023;330(21):2064-2074. doi:10.1001/jama.2023.19918
132. Lu Z, Wang J, Shu Y, et al. Sintilimab versus placebo in combination with chemotherapy as first line treatment for locally advanced or metastatic oesophageal squamous cell carcinoma (ORIENT-15): multicentre, randomised, double blind, phase 3 trial. BMJ. 2022;377:e068714. doi:10.1136/bmj-2021-068714
133. Lu S, Wu L, Jian H, et al. Sintilimab plus bevacizumab biosimilar IBI305 and chemotherapy for patients with EGFR-mutated non-squamous non-small-cell lung cancer who progressed on EGFR tyrosine-kinase inhibitor therapy (ORIENT-31): first interim results from a randomised, double-blind, multicentre, phase 3 trial. Lancet Oncol. 2022;23(9):1167-1179. doi:10.1016/S1470-2045(22)00382-5
134. Cheng Y, Fan Y, Zhao Y, et al. Tislelizumab Plus Platinum and Etoposide Versus Placebo Plus Platinum and Etoposide as First-Line Treatment for Extensive-Stage SCLC (RATIONALE-312): A Multicenter, Double-Blind, Placebo-Controlled, Randomized, Phase 3 Clinical Trial. J Thorac Oncol. 2024;19(7):1073-1085. doi:10.1016/j.jtho.2024.03.008
135. Lu S, Zhang W, Wu L, et al. Perioperative Toripalimab Plus Chemotherapy for Patients With Resectable Non-Small Cell Lung Cancer: The Neotorch Randomized Clinical Trial. JAMA. 2024;331(3):201-211. doi:10.1001/jama.2023.24735
